# Supplementary material for: Global burden of polycystic ovary syndrome in women of reproductive age, 1990–2021: Analysis of the global burden of disease study 2021 with projections to 2050
Source: PLoS One. 2025 Oct 7;20(10):e0333000. doi: 10.1371/journal.pone.0333000 (PMC12503323; doi:10.1371/journal.pone.0333000)
Supplement: S1 Table — (DOCX) [file pone.0333000.s001.docx]

Table 2. Prevalence of PCOS among women of reproductive age between 1990 and 2021 at the global and regional level.

| Location | 1990 | | 2021 | | 1990-2021 EAPC |
| --- | --- | --- | --- | --- | --- |
|  | All-age cases | ASPR per 100,000 population | All-age cases | ASPR per 100,000 population |  |
|  | n*10^3^ (95% UI) | n (95% UI) | n*10^3^ (95% UI) | n (95% UI) | n (95% CI) |
| Global | 34806.51  (24730.06, 48620.13) | 2628.48  (1870.21, 3668.82) | 65767.55  (46839.86, 91498.22) | 3364.53  (2395.08, 4681.81) | 0.74  (0.70, 0.77) |
| SDI |  |  |  |  |  |
| Low SDI | 1059.36  (726.28, 1517.66) | 959.28  (662.55, 1366.05) | 3726.64  (2568.94, 5307.75) | 1369.98  (948.19, 1943.79) | 1.23  (1.20, 1.26) |
| Low-middle SDI | 3864.82  (2682.46, 5463.09) | 1426.32  (994.97, 2010.88) | 11502.69  (8054.04, 16303.71) | 2273.50  (1593.33, 3221.85) | 1.63  (1.59, 1.68) |
| Middle SDI | 10000.31  (7026.35, 13972.04) | 2239.82  (1577.05, 3125.69) | 23243.05  (16338.14, 32445.09) | 3758.81  (2638.95, 5246.88) | 1.73  (1.69, 1.78) |
| High-middle SDI | 6686.76  (4739.05, 9288.98) | 2408.19  (1707.28, 3345.96) | 10547.25  (7422.93, 14799.94) | 3466.45  (2432.25, 4866.13) | 1.21  (1.17, 1.25) |
| High SDI | 13171.90  (9494.10, 18373.08) | 5761.19  (4149.09, 8038.07) | 16702.14  (12287.90, 22846.79) | 6825.02  (5014.42, 9336.43) | 0.10  (-0.08, 0.27) |
| Regions |  |  |  |  |  |
| East Asia | 5386.01  (3728.94, 7623.47) | 1624.24  (1126.80, 2294.93) | 9873.93  (6871.37, 14027.82) | 2967.58  (2057.24, 4219.21) | 2.04  (1.88, 2.19) |
| Southeast Asia | 3506.30  (2439.74, 5011.79) | 2928.59  (2040.79, 4182.23) | 9998.31  (7018.88, 14135.47) | 5444.39  (3819.23, 7698.36) | 2.30  (2.19, 2.40) |
| Oceania | 37.97  (26.13, 54.21) | 2446.04  (1687.00, 3491.16) | 117.78  (80.92, 168.38) | 3384.68  (2326.80, 4838.62) | 0.81  (0.64, 0.99) |
| Central Asia | 112.00  (74.34, 163.73) | 665.96  (443.93, 971.29) | 225.49  (152.30, 319.48) | 923.53  (622.33, 1311.00) | 1.18  (1.11, 1.25) |

(continued)

Table 2 Continued

| Location | 1990 | | 2021 | | 1990-2021 EAPC |
| --- | --- | --- | --- | --- | --- |
|  | All-age cases | ASPR per 100,000 population | All-age cases | ASPR per 100,000 population |  |
|  | n*10^3^ (95% UI) | n (95% UI) | n*10^3^ (95% UI) | n (95% UI) | n (95% CI) |
| Central Europe | 109.04  (71.06, 163.32) | 353.27  (229.55, 530.29) | 112.35  (77.04, 158.86) | 436.26  (297.37, 620.93) | 0.64  (0.59, 0.70) |
| Eastern Europe | 221.99  (148.93, 323.03) | 394.71  (263.68, 577.44) | 251.01  (172.88, 364.63) | 507.61  (344.81, 744.99) | 0.99  (0.94, 1.04) |
| High-income Asia Pacific | 4201.47  (2942.53, 5939.04) | 9136.96  (6399.84, 12907.51) | 3894.79  (2730.35, 5504.21) | 10116.87  (7086.92, 14260.97) | 0.27  (0.22, 0.32) |
| Australasia | 425.03  (305.67, 573.28) | 7885.92  (5669.35, 10638.01) | 665.14  (468.24, 929.03) | 9156.94  (6439.94, 12788.23) | 0.27  (0.18, 0.37) |
| Western Europe | 6457.42  (4532.42, 8980.93) | 6728.43  (4718.01, 9363.55) | 7005.90  (4913.70, 9719.71) | 7493.55  (5251.94, 10400.43) | 0.21  (0.14, 0.28) |
| Southern Latin America | 282.12  (195.49, 407.70) | 2281.39  (1581.15, 3295.45) | 637.61  (443.68, 918.53) | 3637.79  (2531.50, 5240.58) | 1.46  (1.24, 1.69) |
| High-income North America | 4287.62  (3007.14, 6077.37) | 5695.27  (3989.30, 8080.87) | 6071.38  (4473.19, 8127.16) | 7200.01  (5306.10, 9639.58) | -0.50  (-1.01, 0.01) |
| Caribbean | 210.46  (141.90, 301.26) | 2256.95  (1525.39, 3228.82) | 339.90  (230.31, 486.51) | 2823.51  (1912.23, 4041.39) | 0.76  (0.70, 0.83) |
| Andean Latin America | 433.32  (293.65, 612.91) | 4572.25  (3099.89, 6466.11) | 1105.17  (756.38, 1574.80) | 6305.75  (4312.97, 8986.98) | 1.09  (1.01, 1.16) |
| Central Latin America | 2128.22  (1459.24, 3001.88) | 5067.18  (3477.64, 7147.64) | 3806.92  (2643.27, 5352.32) | 5580.86  (3873.19, 7846.44) | -0.09  (-0.26, 0.08) |
| Tropical Latin America | 418.59  (281.62, 610.84) | 1049.41  (709.10, 1528.59) | 694.30  (473.82, 991.60) | 1140.94  (776.47, 1632.49) | -0.17  (-0.35, 0.00) |

(continued)

Table 2 Continued

| Location | 1990 | | 2021 | | 1990-2021 EAPC |
| --- | --- | --- | --- | --- | --- |
|  | All-age cases | ASPR per 100,000 population | All-age cases | ASPR per 100,000 population |  |
|  | n*10^3^ (95% UI) | n (95% UI) | n*10^3^ (95% UI) | n (95% UI) | n (95% CI) |
| North Africa and Middle East | 2314.90  (1597.57, 3312.21) | 2963.30  (2050.08, 4236.92) | 6335.26  (4411.19, 9007.27) | 3968.60  (2762.78, 5641.91) | 1.09  (1.02, 1.16) |
| South Asia | 3111.98  (2196.91, 4339.79) | 1229.14  (870.96, 1709.54) | 10749.37  (7552.17, 15115.14) | 2174.75  (1528.67, 3058.06) | 2.14  (2.01, 2.27) |
| Central Sub-Saharan Africa | 104.33  (70.08, 151.99) | 854.52  (578.18, 1239.36) | 418.51  (284.03, 605.62) | 1291.50  (880.09, 1861.86) | 1.28  (1.13, 1.43) |
| Eastern Sub-Saharan Africa | 422.76  (288.22, 606.43) | 990.93  (682.45, 1412.06) | 1362.79  (935.96, 1948.40) | 1282.62  (885.52, 1826.00) | 0.86  (0.83, 0.89) |
| Southern Sub-Saharan Africa | 220.25  (150.00, 318.03) | 1667.09  (1139.60, 2398.24) | 456.80  (312.38, 653.58) | 2094.17  (1431.86, 2995.98) | 0.79  (0.70, 0.87) |
| Western Sub-Saharan Africa | 414.74  (282.55, 598.44) | 962.89  (662.22, 1378.92) | 1644.81  (1124.12, 2347.99) | 1385.41  (951.70, 1970.02) | 0.92  (0.74, 1.11) |

Abbreviations: ASPR, age standardized prevalence rate; EAPC, estimated annual percentage change; SDI, socio-demographic index; UI, uncertainty interval; CI, confidence interval
