# Supplementary material for: Global burden of polycystic ovary syndrome in women of reproductive age, 1990–2021: Analysis of the global burden of disease study 2021 with projections to 2050
Source: PLoS One. 2025 Oct 7;20(10):e0333000. doi: 10.1371/journal.pone.0333000 (PMC12503323; doi:10.1371/journal.pone.0333000)
Supplement: S2 Table — (DOCX) [file pone.0333000.s002.docx]

Table 3. DALYs of PCOS among women of reproductive age between 1990 and 2021 at the global and regional level.

| Location | 1990 | | 2021 | | 1990-2021 EAPC |
| --- | --- | --- | --- | --- | --- |
|  | All-age DALYs | ASDR per 100,000 population | All-age DALYs | ASDR per 100,000 population |  |
|  | n*10^3^ (95% UI) | n (95% UI) | n*10^3^ (95% UI) | n (95% UI) | n (95% CI) |
| Global | 307.94  (136.41, 644.50) | 23.16  (10.27, 48.43) | 576.05  (255.58, 1200.18) | 29.51  (13.09, 61.49) | 0.72  (0.68, 0.76) |
| SDI |  |  |  |  |  |
| Low SDI | 9.30  (3.96, 19.59) | 8.35  (3.56, 17.58) | 32.61  (13.96, 68.77) | 11.91  (5.10, 25.15) | 1.21  (1.19, 1.24) |
| Low-middle SDI | 34.54  (14.84, 72.78) | 12.65  (5.43, 26.64) | 101.24  (43.89, 212.88) | 19.97  (8.66, 41.98) | 1.59  (1.55, 1.63) |
| Middle SDI | 88.35  (38.31, 186.88) | 19.67  (8.53, 41.61) | 203.16  (88.68, 427.66) | 32.95  (14.39, 69.38) | 1.72  (1.67, 1.77) |
| High-middle SDI | 58.69  (25.89, 123.52) | 21.10  (9.31, 44.40) | 91.76  (40.39, 193.25) | 30.38  (13.37, 64.03) | 1.20  (1.16, 1.25) |
| High SDI | 116.85  (52.34, 242.34) | 51.16  (22.91, 106.02) | 146.88  (66.64, 298.93) | 60.32  (27.37, 122.77) | 0.08  (-0.09, 0.25) |
| Regions |  |  |  |  |  |
| East Asia | 46.32  (19.91, 97.55) | 13.91  (5.99, 29.33) | 84.71  (36.82, 177.52) | 25.64  (11.15, 53.82) | 2.05  (1.89, 2.22) |
| Southeast Asia | 31.34  (13.51, 63.91) | 26.01  (11.22, 53.02) | 88.13  (38.49, 182.97) | 48.06  (20.99, 99.85) | 2.26  (2.16, 2.36) |
| Oceania | 0.33  (0.14, 0.70) | 21.32  (9.27, 45.06) | 1.03  (0.44, 2.14) | 29.44  (12.73, 61.41) | 0.81  (0.64, 0.99) |
| Central Asia | 0.99  (0.41, 2.11) | 5.86  (2.42, 12.49) | 1.97  (0.83, 4.23) | 8.10  (3.40, 17.41) | 1.16  (1.09, 1.23) |

(continued)

Table 3. Continued

| Location | 1990 | | 2021 | | 1990-2021 EAPC |
| --- | --- | --- | --- | --- | --- |
|  | All-age cases | ASDR per 100,000 population | All-age cases | ASDR per 100,000 population |  |
|  | n*10^3^ (95% UI) | n (95% UI) | n*10^3^ (95% UI) | n (95% UI) | n (95% CI) |
| Central Europe | 0.95  (0.40, 2.00) | 3.10  (1.28, 6.51) | 0.97  (0.41, 2.08) | 3.81  (1.62, 8.15) | 0.64  (0.59, 0.69) |
| Eastern Europe | 1.97  (0.81, 4.17) | 3.51  (1.44, 7.46) | 2.20  (0.91, 4.62) | 4.51  (1.86, 9.53) | 0.98  (0.93, 1.03) |
| High-income Asia Pacific | 36.55  (16.04, 74.84) | 79.69  (34.97, 163.05) | 33.72  (15.06, 69.11) | 88.17  (39.21, 180.74) | 0.27  (0.22, 0.31) |
| Australasia | 3.71  (1.64, 7.61) | 68.88  (30.52, 141.27) | 5.79  (2.57, 12.09) | 79.97  (35.50, 166.66) | 0.28  (0.19, 0.37) |
| Western Europe | 57.72  (25.92, 120.53) | 60.15  (26.99, 125.73) | 62.19  (27.84, 129.96) | 66.97  (29.95, 140.01) | 0.22  (0.15, 0.28) |
| Southern Latin America | 2.51  (1.08, 5.18) | 20.23  (8.74, 41.87) | 5.64  (2.44, 11.72) | 32.22  (13.93, 66.95) | 1.46  (1.24, 1.68) |
| High-income North America | 38.42  (16.83, 78.96) | 51.04  (22.36, 104.77) | 53.69  (24.48, 107.31) | 63.85  (29.11, 127.64) | -0.51  (-1.02, -0.01) |
| Caribbean | 1.88  (0.80, 3.99) | 20.01  (8.57, 42.44) | 2.98  (1.28, 6.31) | 24.79  (10.61, 52.51) | 0.75  (0.68, 0.82) |
| Andean Latin America | 3.77  (1.66, 8.13) | 39.67  (17.47, 85.41) | 9.55  (4.20, 20.09) | 54.46  (23.94, 114.59) | 1.06  (0.99, 1.14) |
| Central Latin America | 18.71  (8.24, 39.65) | 44.32  (19.46, 93.84) | 33.03  (14.40, 69.72) | 48.44  (21.12, 102.24) | -0.10  (-0.27, 0.06) |
| Tropical Latin America | 3.75  (1.57, 8.06) | 9.37  (3.92, 20.09) | 6.11  (2.60, 13.09) | 10.08  (4.28, 21.62) | -0.19  (-0.37, -0.01) |

(continued)

Table 3. Continued

| Location | 1990 | | 2021 | | 1990-2021 EAPC |
| --- | --- | --- | --- | --- | --- |
|  | All-age cases | ASDR per 100,000 population | All-age cases | ASDR per 100,000 population |  |
|  | n*10^3^ (95% UI) | n (95% UI) | n*10^3^ (95% UI) | n (95% UI) | n (95% CI) |
| North Africa and Middle East | 21.11  (9.22, 44.34) | 26.79  (11.72, 56.26) | 56.22  (24.68, 118.45) | 35.25  (15.48, 74.26) | 1.04  (0.96, 1.11) |
| South Asia | 27.78  (12.08, 58.75) | 10.89  (4.73, 23.04) | 94.33  (40.62, 197.25) | 19.06  (8.21, 39.87) | 2.08  (1.97, 2.20) |
| Central Sub-Saharan Africa | 0.91  (0.37, 1.91) | 7.35  (3.04, 15.49) | 3.64  (1.55, 7.65) | 11.15  (4.74, 23.40) | 1.27  (1.11, 1.44) |
| Eastern Sub-Saharan Africa | 3.69  (1.56, 7.73) | 8.56  (3.63, 17.95) | 11.84  (5.07, 25.04) | 11.07  (4.74, 23.42) | 0.85  (0.82, 0.88) |
| Southern Sub-Saharan Africa | 1.94  (0.84, 4.15) | 14.56  (6.29, 31.06) | 3.96  (1.69, 8.43) | 18.14  (7.73, 38.63) | 0.75  (0.67, 0.83) |
| Western Sub-Saharan Africa | 3.61  (1.53, 7.63) | 8.30  (3.53, 17.50) | 14.34  (6.11, 30.46) | 11.98  (5.11, 25.47) | 0.92  (0.74, 1.11) |

Abbreviations: ASDR, age standardized DALYs rate; EAPC, estimated annual percentage change; SDI, socio-demographic index; UI, uncertainty interval; CI, confidence interval
