# Supplementary figures and images for: Global burden of polycystic ovary syndrome in women of reproductive age, 1990–2021: Analysis of the global burden of disease study 2021 with projections to 2050
Source: PLoS One. 2025 Oct 7;20(10):e0333000. doi: 10.1371/journal.pone.0333000 (PMC12503323; doi:10.1371/journal.pone.0333000)

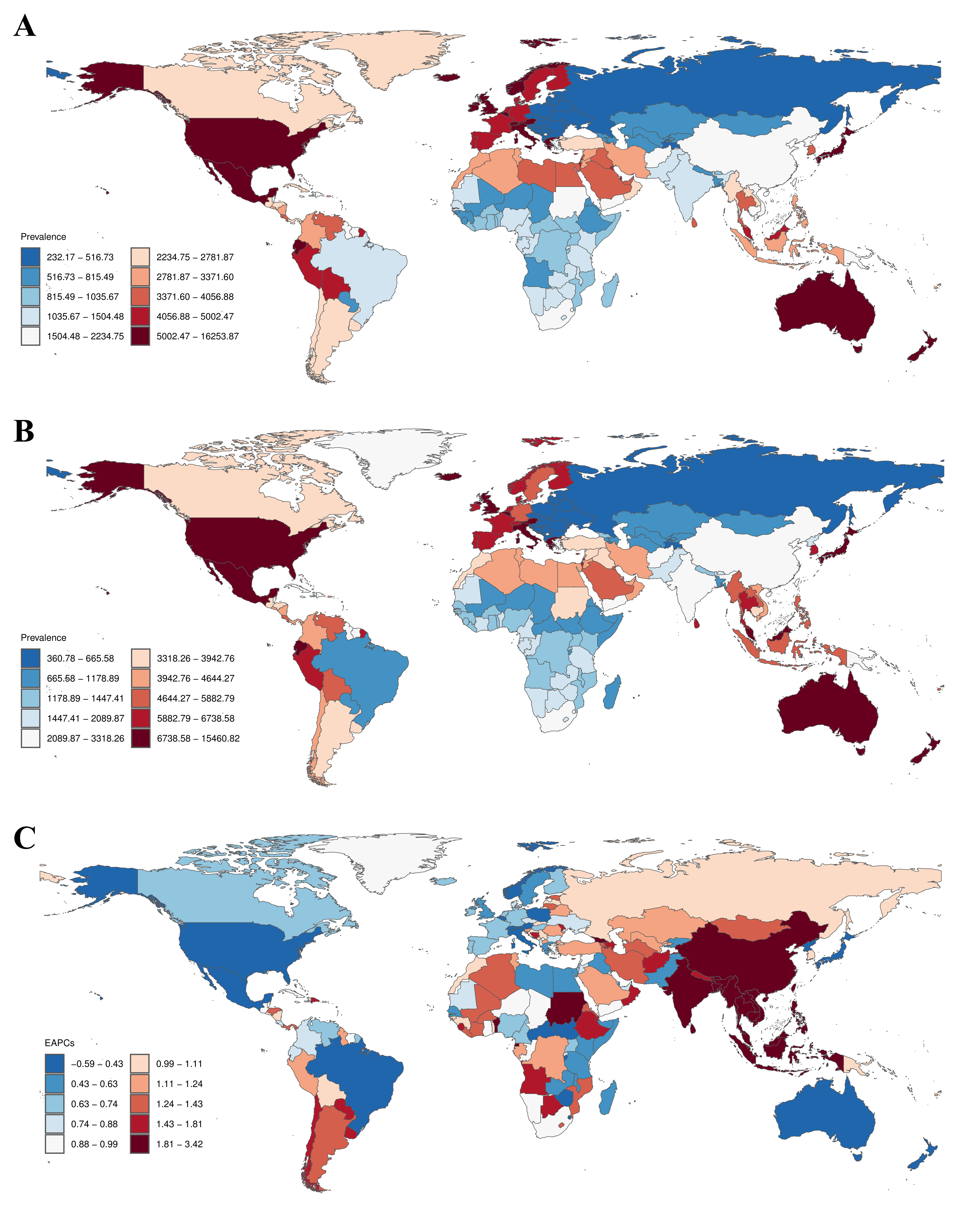

Supplement: S2 Fig — (A) ASPR in 1990. (B) ASPR in 2021. (C) EAPC between 1990 and 2021. (TIF) [file pone.0333000.s004.tif]

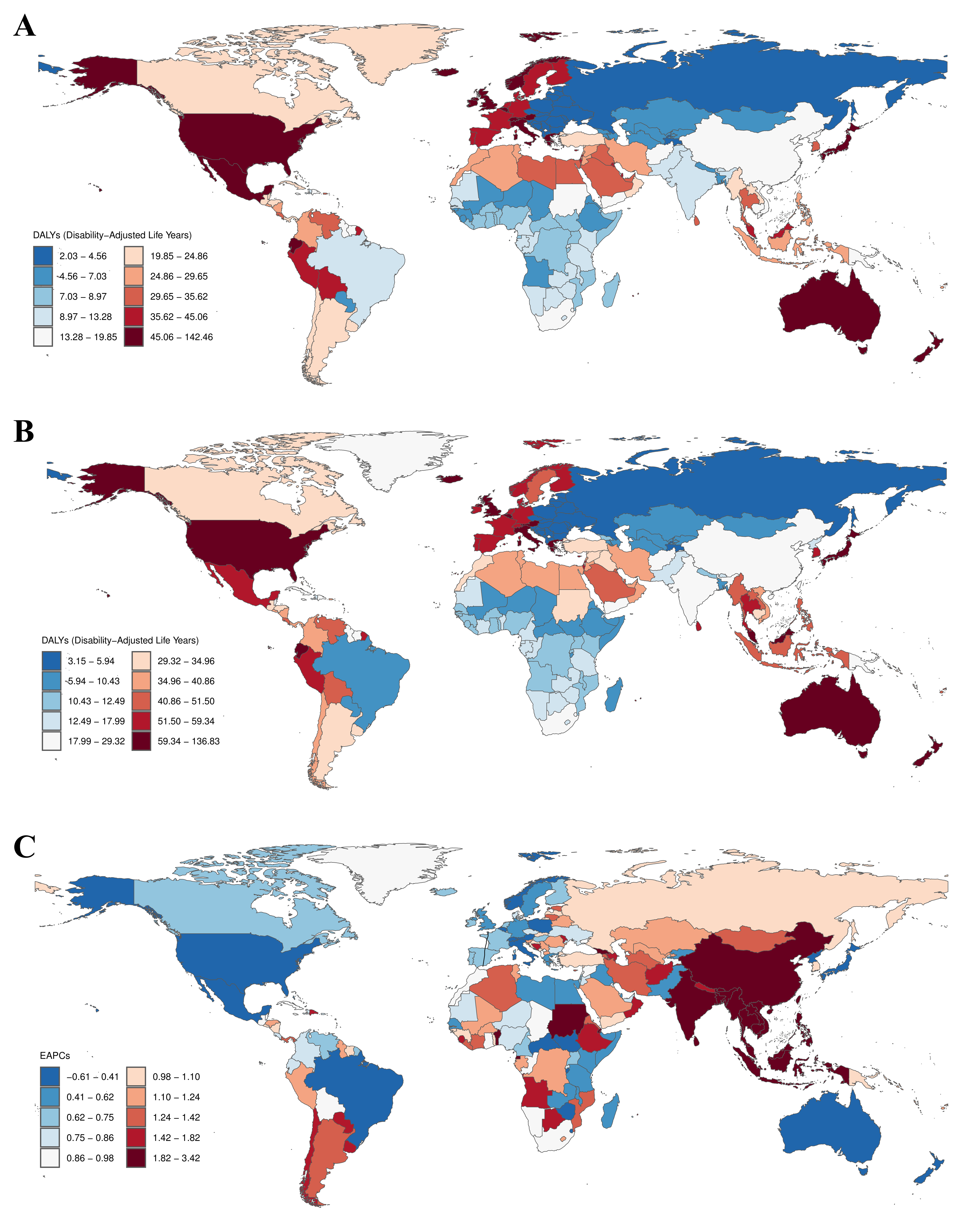

Supplement: S4 Fig — (A) ASDR in 1990. (B) ASDR in 2021. (C) EAPC between 1990 and 2021. (TIF) [file pone.0333000.s006.tif]

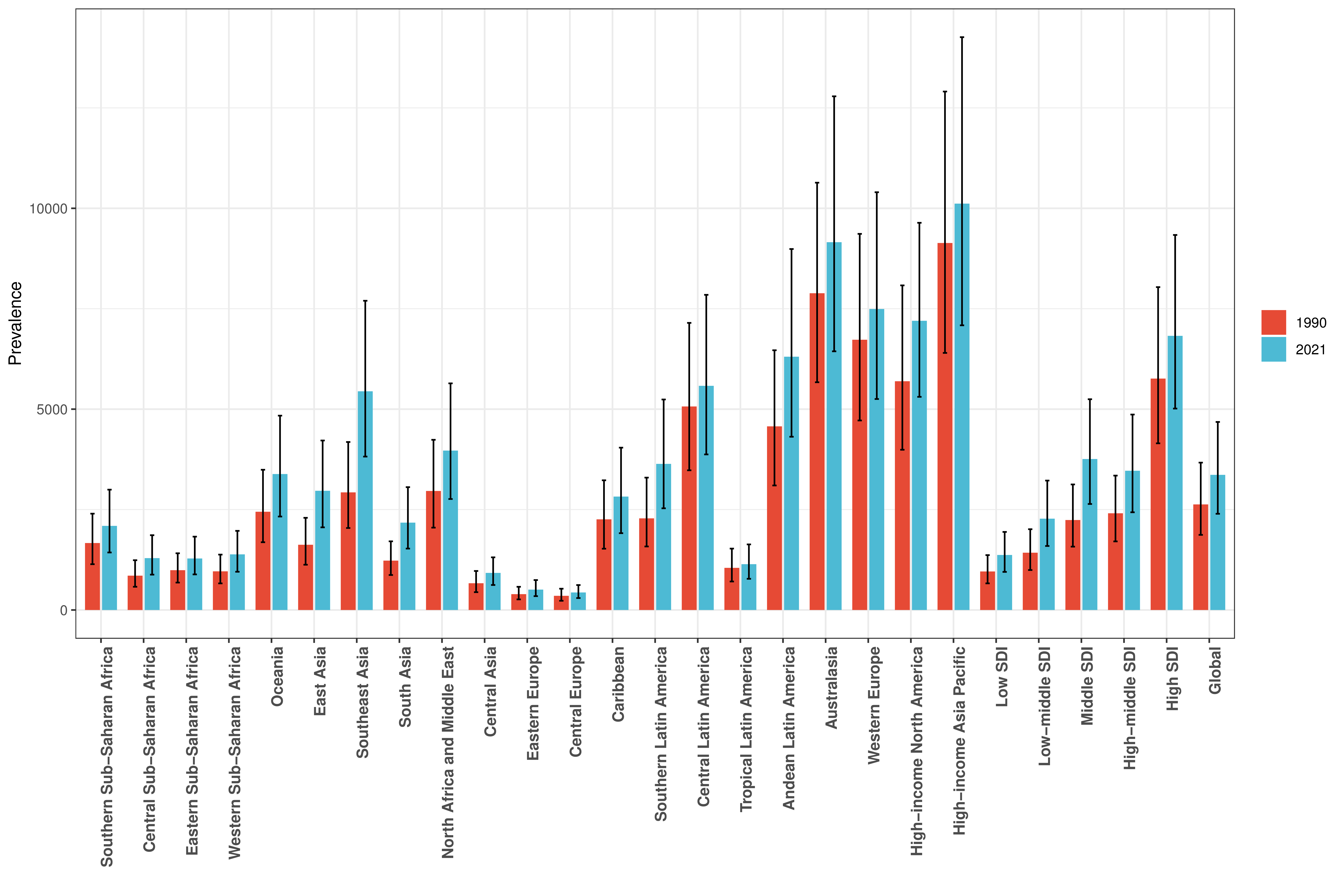

Supplement: S6 Fig — (TIF) [file pone.0333000.s008.tif]

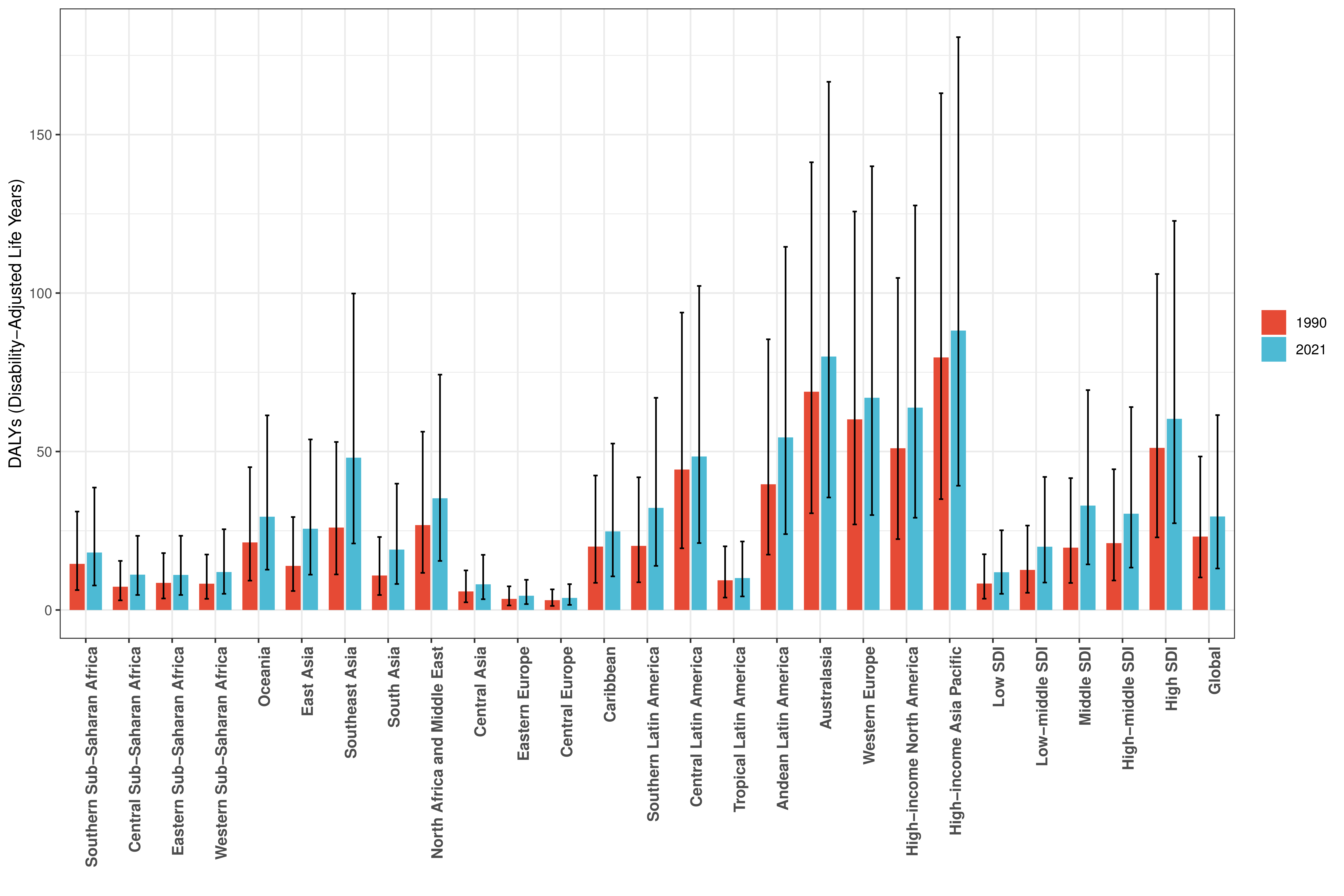

Supplement: S7 Fig — (TIF) [file pone.0333000.s009.tif]

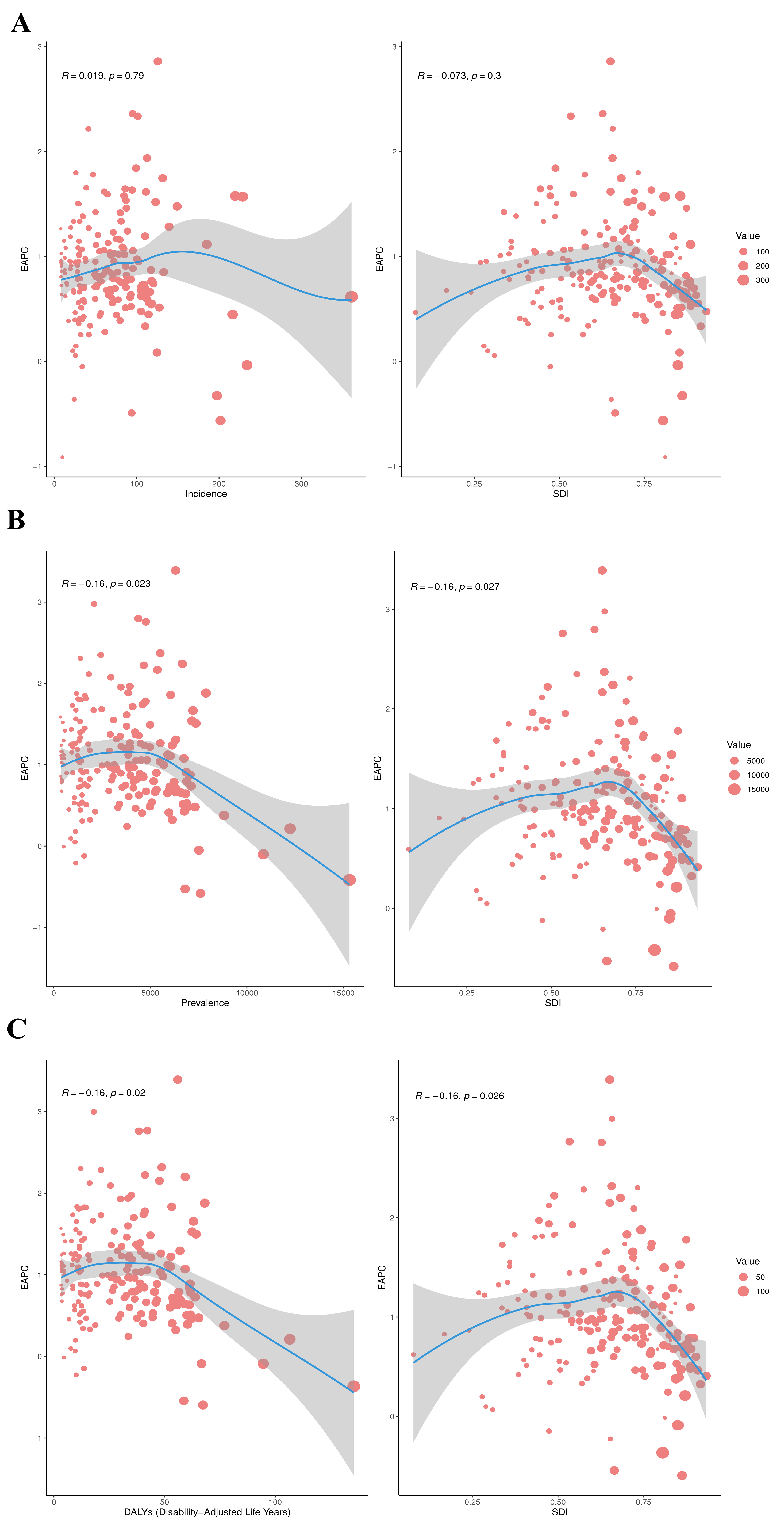

Supplement: S8 Fig — (A) ASIR. (B) ASPR. (C) ASDR. (TIF) [file pone.0333000.s010.tif]

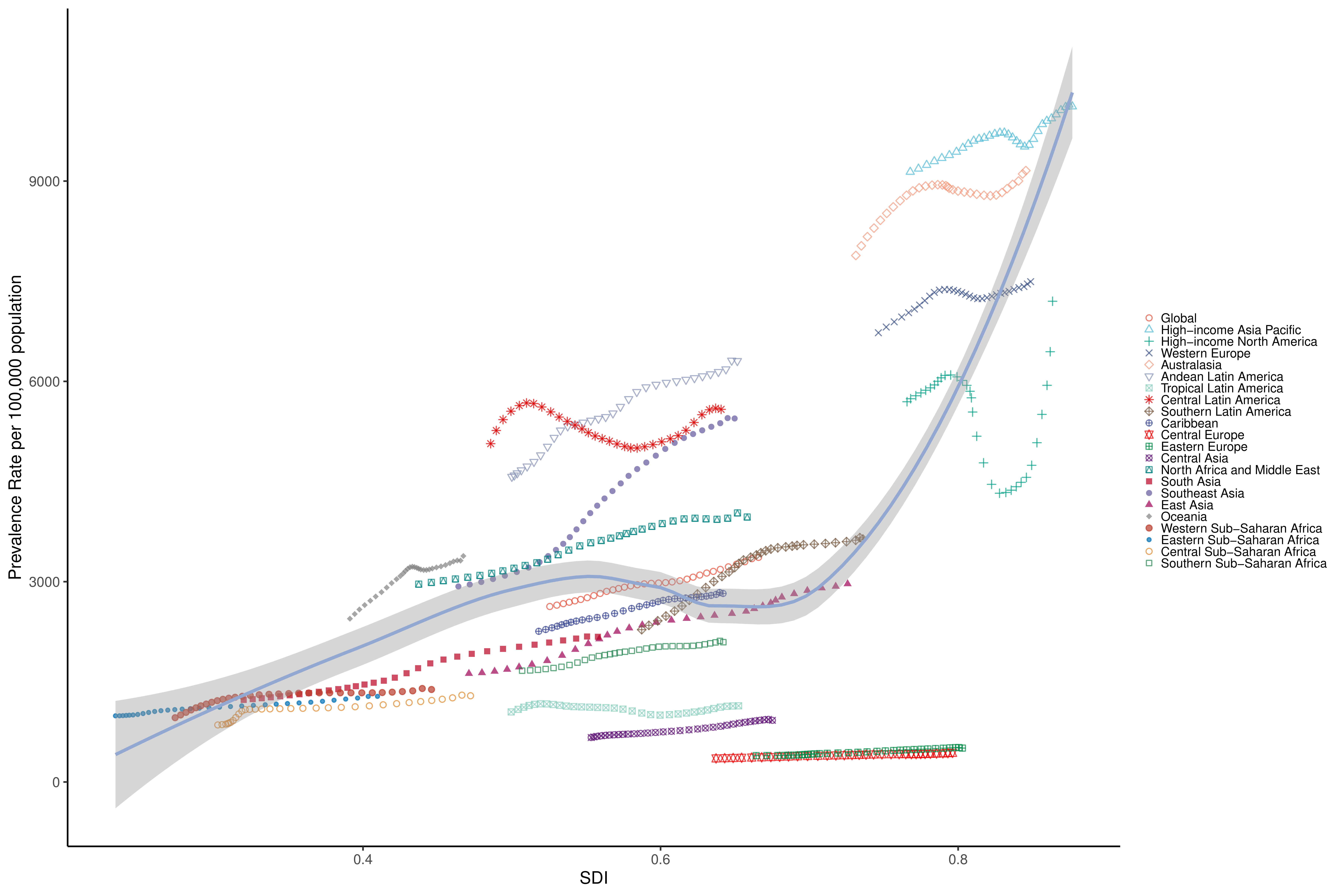

Supplement: S9 Fig — (TIF) [file pone.0333000.s011.tif]

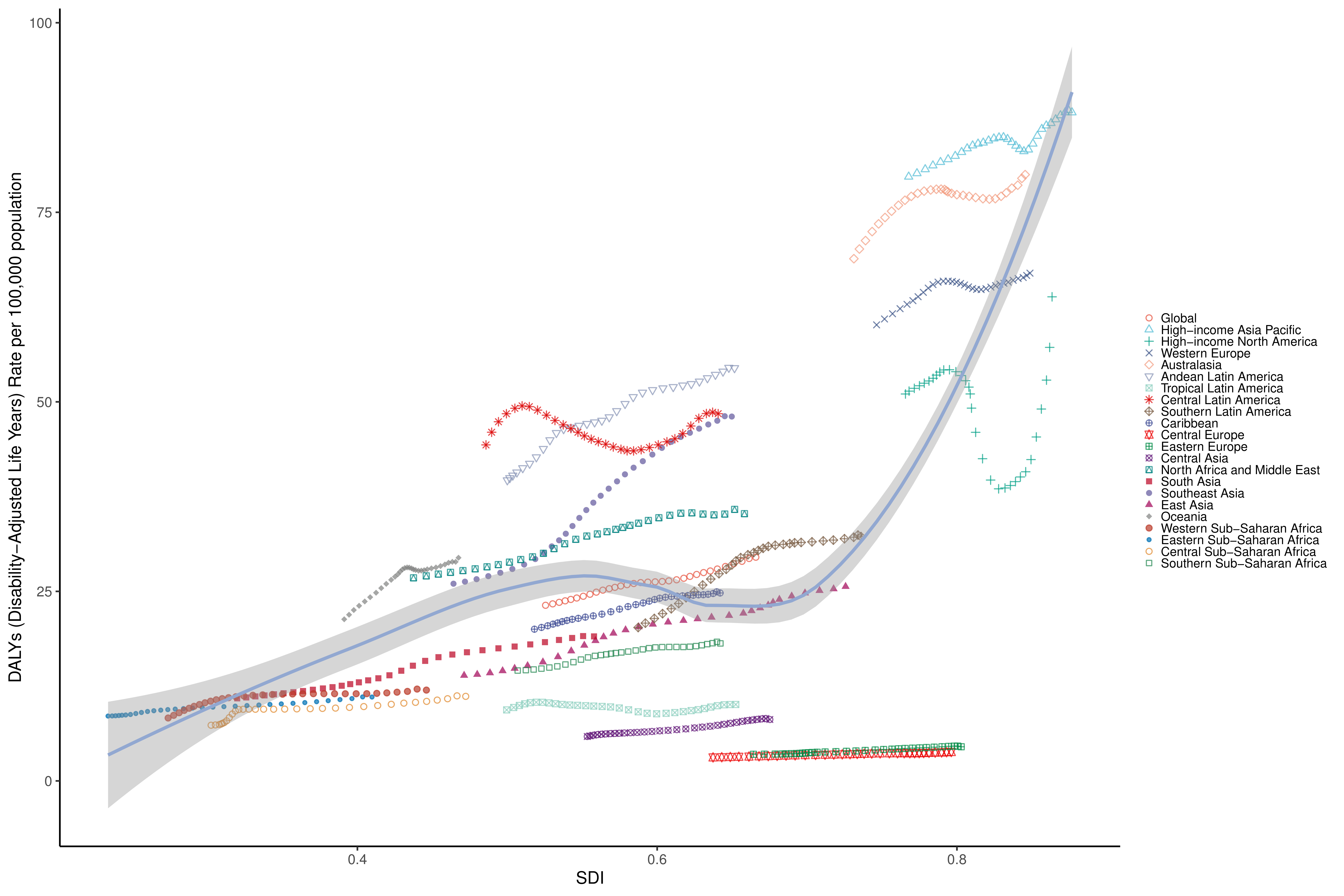

Supplement: S10 Fig — (TIF) [file pone.0333000.s012.tif]
