# Supplementary material for: Global burden of polycystic ovary syndrome in women of reproductive age, 1990–2021: Analysis of the global burden of disease study 2021 with projections to 2050
Source: PLoS One. 2025 Oct 7;20(10):e0333000. doi: 10.1371/journal.pone.0333000 (PMC12503323; doi:10.1371/journal.pone.0333000)
Supplement: S3 Table — (DOCX) [file pone.0333000.s013.docx]

Table 4. Incidence of PCOS among women of reproductive age between 1990 and 2021 at the national level.

| Nations | 1990 | | 2021 | | 1990-2021 EAPC |
| --- | --- | --- | --- | --- | --- |
|  | All-age cases | ASIR per 100,000 population | All-age cases | ASIR per 100,000 population |  |
|  | n (95% UI) | n (95% UI) | n (95% UI) | n (95% UI) | n (95% CI) |
| American Samoa | 9.93  (16.74, 6.18) | 70.67  (118.93, 43.92) | 13.01  (21.57, 8.27) | 92.92  (154.55, 58.83) | 0.78  (0.63, 0.94) |
| Antigua and Barbuda | 6.87  (11.40, 4.24) | 40.31  (66.75, 24.90) | 9.90  (16.49, 6.18) | 51.35  (85.09, 32.29) | 0.71  (0.67, 0.75) |
| Arab Republic of Egypt | 10314.69  (17407.61, 6424.01) | 63.38  (107.23, 39.28) | 21416.57  (35655.80, 13486.46) | 77.44  (128.96, 48.72) | 0.59  (0.54, 0.63) |
| Argentine Republic | 4621.63  (7763.28, 2660.97) | 53.72  (90.39, 30.87) | 8486.29  (14016.92, 4942.13) | 81.04  (133.57, 47.31) | 1.34  (1.17, 1.50) |
| Australia | 7357.83  (11952.38, 4374.79) | 181.13  (294.09, 107.76) | 9399.66  (15582.01, 5470.26) | 216.47  (358.38, 126.22) | 0.45  (0.39, 0.50) |
| Barbados | 31.98  (53.34, 19.96) | 47.70  (79.35, 29.85) | 31.60  (51.37, 19.67) | 55.19  (89.39, 34.59) | 0.44  (0.40, 0.47) |
| Belize | 24.33  (40.84, 15.02) | 40.51  (68.32, 24.76) | 75.01  (122.31, 47.68) | 54.91  (89.64, 34.80) | 0.82  (0.62, 1.02) |
| Bermuda | 6.71  (11.09, 4.15) | 55.13  (90.52, 34.53) | 5.65  (8.73, 3.61) | 61.18  (94.04, 39.54) | 0.28  (0.23, 0.33) |
| Bolivarian Republic of Venezuela | 3275.47  (5051.94, 2117.68) | 56.11  (86.71, 36.15) | 4471.28  (6839.29, 2986.84) | 69.62  (106.76, 46.49) | 0.61  (0.56, 0.66) |
| Bosnia and Herzegovina | 62.93  (112.85, 33.25) | 5.52  (9.87, 2.93) | 43.03  (76.04, 22.79) | 7.84  (13.68, 4.28) | 1.26  (1.10, 1.42) |
| Brunei Darussalam | 102.29  (163.09, 58.34) | 144.27  (229.79, 82.30) | 220.44  (351.03, 120.75) | 228.76  (363.60, 125.51) | 1.57  (1.40, 1.74) |
| Burkina Faso | 563.87  (969.44, 318.90) | 20.90  (36.15, 11.63) | 2139.45  (3639.10, 1239.99) | 29.53  (50.49, 16.91) | 0.95  (0.80, 1.10) |
| Canada | 3803.65  (6262.35, 2211.24) | 67.57  (110.79, 39.48) | 5257.72  (8497.25, 3111.32) | 87.47  (140.96, 51.96) | 0.82  (0.70, 0.94) |
| Central African Republic | 191.72  (323.49, 110.52) | 23.91  (40.43, 13.63) | 451.15  (770.88, 265.84) | 25.56  (43.92, 14.87) | 0.06  (-0.04, 0.15) |
| Commonwealth of Dominica | 9.00  (14.99, 5.51) | 40.27  (67.38, 24.46) | 8.67  (13.90, 5.42) | 52.29  (83.86, 32.63) | 0.73  (0.64, 0.82) |
| Commonwealth of the Bahamas | 38.62  (65.23, 24.49) | 48.07  (81.07, 30.44) | 57.95  (93.88, 36.40) | 56.44  (91.46, 35.47) | 0.46  (0.40, 0.52) |
| Cook Islands | 4.14  (6.86, 2.60) | 72.37  (120.22, 45.36) | 4.33  (7.18, 2.78) | 100.32  (166.10, 64.30) | 0.97  (0.86, 1.09) |
| Czech Republic | 162.20  (284.94, 89.39) | 6.68  (11.76, 3.68) | 130.90  (228.81, 72.16) | 8.31  (14.39, 4.71) | 0.72  (0.67, 0.77) |
| Democratic People's Republic of Korea | 2282.38  (3907.47, 1329.98) | 37.69  (64.31, 21.97) | 2286.41  (3813.25, 1352.42) | 41.73  (69.14, 24.88) | 0.26  (0.18, 0.33) |
| Democratic Republic of Sao Tome and Principe | 8.64  (15.05, 4.97) | 23.65  (41.54, 13.35) | 22.12  (37.73, 12.81) | 31.75  (54.41, 18.20) | 0.79  (0.64, 0.94) |
| Democratic Republic of the Congo | 2303.96  (3993.51, 1299.08) | 20.81  (36.26, 11.56) | 7738.54  (13121.33, 4502.03) | 27.71  (47.31, 15.90) | 0.91  (0.79, 1.04) |
| Democratic Republic of Timor-Leste | 120.53  (202.99, 71.38) | 58.20  (97.80, 34.45) | 407.58  (669.15, 246.89) | 86.01  (141.56, 51.86) | 1.64  (1.53, 1.76) |
| Democratic Socialist Republic of Sri Lanka | 4211.06  (7091.08, 2395.37) | 83.50  (140.63, 47.44) | 6566.40  (11056.63, 3886.04) | 122.93  (207.02, 72.77) | 1.52  (1.29, 1.75) |
| Dominican Republic | 895.83  (1497.98, 565.24) | 36.72  (61.54, 23.00) | 1500.11  (2454.13, 940.49) | 52.92  (86.37, 33.23) | 1.20  (1.14, 1.27) |
| Eastern Republic of Uruguay | 420.58  (701.75, 246.89) | 55.35  (92.40, 32.47) | 623.85  (1036.72, 362.95) | 86.89  (144.13, 50.67) | 1.53  (1.36, 1.71) |
| Federal Democratic Republic of Ethiopia | 3049.67  (5229.33, 1729.72) | 21.56  (37.00, 12.07) | 10241.60  (17438.30, 5922.06) | 28.74  (49.09, 16.43) | 1.08  (1.03, 1.14) |
| Federal Democratic Republic of Nepal | 1083.33  (1898.15, 605.66) | 19.42  (34.12, 10.74) | 2890.44  (4849.31, 1679.05) | 29.75  (49.78, 17.28) | 1.50  (1.46, 1.55) |
| Federal Republic of Germany | 11184.41  (17286.07, 7062.70) | 85.82  (131.24, 54.67) | 12146.74  (19385.89, 7698.88) | 105.75  (168.06, 67.42) | 0.63  (0.58, 0.68) |
| Federal Republic of Nigeria | 7396.95  (12615.09, 4268.85) | 26.71  (45.70, 15.19) | 25641.63  (43493.72, 14937.52) | 33.14  (56.53, 19.06) | 0.51  (0.39, 0.63) |
| Federal Republic of Somalia | 483.43  (826.73, 274.93) | 22.43  (38.81, 12.54) | 1662.11  (2808.51, 964.51) | 25.14  (42.75, 14.36) | 0.47  (0.43, 0.50) |
| Federated States of Micronesia | 18.73  (30.72, 11.49) | 56.47  (93.18, 34.35) | 23.85  (38.50, 14.96) | 76.62  (123.87, 47.90) | 0.85  (0.68, 1.02) |
| Federative Republic of Brazil | 10515.66  (18204.98, 6361.31) | 23.52  (40.73, 14.12) | 11779.63  (19941.68, 7006.19) | 24.06  (40.50, 14.53) | -0.36  (-0.58, -0.15) |
| French Republic | 11025.97  (18115.71, 6934.06) | 84.96  (139.44, 53.52) | 12704.80  (20460.25, 8094.17) | 104.92  (168.85, 66.95) | 0.68  (0.63, 0.73) |
| Gabonese Republic | 81.85  (145.73, 47.57) | 27.44  (48.95, 15.73) | 221.57  (388.40, 127.81) | 37.86  (66.47, 21.71) | 0.96  (0.84, 1.07) |
| Georgia | 212.24  (367.57, 118.29) | 16.73  (28.79, 9.40) | 146.68  (254.84, 86.89) | 26.07  (45.03, 15.73) | 1.80  (1.60, 2.00) |
| Grand Duchy of Luxembourg | 60.86  (97.42, 38.49) | 93.02  (147.97, 59.31) | 116.31  (186.38, 74.70) | 118.11  (188.49, 76.29) | 0.77  (0.70, 0.83) |
| Greenland | 6.22  (10.26, 3.58) | 58.48  (95.11, 34.04) | 8.52  (13.97, 4.99) | 78.03  (127.75, 45.80) | 1.02  (0.91, 1.13) |
| Grenada | 9.09  (14.93, 5.55) | 36.23  (59.74, 21.92) | 11.42  (18.56, 7.22) | 48.39  (78.20, 30.75) | 0.83  (0.73, 0.93) |
| Guam | 26.56  (45.02, 16.10) | 75.97  (128.56, 46.12) | 35.15  (59.47, 21.95) | 102.07  (172.59, 63.77) | 0.95  (0.87, 1.03) |
| Hashemite Kingdom of Jordan | 746.23  (1247.98, 456.80) | 58.85  (98.80, 35.71) | 2634.96  (4330.97, 1629.88) | 72.27  (119.06, 44.56) | 0.79  (0.74, 0.84) |
| Hellenic Republic | 2192.80  (3566.16, 1393.05) | 95.31  (154.81, 60.63) | 1769.94  (2792.79, 1136.88) | 117.81  (185.52, 75.95) | 0.54  (0.38, 0.70) |
| Hungary | 158.56  (282.35, 88.37) | 7.00  (12.45, 3.92) | 129.17  (228.64, 70.72) | 8.53  (14.91, 4.83) | 0.64  (0.58, 0.70) |
| Independent State of Papua New Guinea | 544.01  (882.65, 330.55) | 44.97  (73.14, 27.16) | 1712.95  (2805.36, 1058.06) | 61.91  (101.31, 38.22) | 0.86  (0.72, 1.00) |
| Independent State of Samoa | 35.57  (59.44, 21.53) | 65.08  (109.37, 39.09) | 50.37  (85.56, 31.58) | 83.73  (142.55, 52.31) | 0.75  (0.64, 0.86) |
| Ireland | 932.16  (1517.25, 593.31) | 92.89  (151.56, 59.00) | 1082.23  (1758.30, 690.19) | 113.86  (184.92, 72.73) | 0.66  (0.57, 0.74) |
| Islamic Republic of Afghanistan | 1330.70  (2185.50, 801.90) | 37.07  (61.79, 21.89) | 5039.44  (8407.00, 3095.13) | 49.94  (83.64, 30.38) | 1.42  (1.19, 1.66) |
| Islamic Republic of Iran | 11013.38  (18292.96, 6691.96) | 63.34  (105.67, 38.20) | 13523.06  (22462.43, 8266.18) | 79.60  (132.07, 48.98) | 1.16  (0.87, 1.45) |
| Islamic Republic of Mauritania | 160.38  (282.18, 91.93) | 26.33  (46.50, 14.90) | 479.74  (830.10, 280.84) | 33.94  (59.00, 19.66) | 0.58  (0.44, 0.72) |
| Islamic Republic of Pakistan | 9802.29  (16792.79, 5663.03) | 31.37  (54.06, 17.89) | 23896.27  (40950.60, 13588.28) | 35.54  (60.91, 20.15) | 0.59  (0.50, 0.67) |
| Jamaica | 296.89  (483.90, 182.90) | 39.10  (63.89, 23.90) | 346.04  (575.72, 209.99) | 50.16  (83.01, 30.60) | 0.80  (0.75, 0.86) |
| Japan | 92031.24  (156230.40, 48555.41) | 307.87  (522.76, 162.44) | 60883.73  (96873.36, 33536.08) | 360.92  (573.59, 199.08) | 0.62  (0.48, 0.75) |
| Kingdom of Bahrain | 79.54  (133.89, 49.12) | 70.71  (118.47, 43.84) | 223.00  (375.61, 136.67) | 79.89  (134.23, 49.15) | 0.39  (0.35, 0.43) |
| Kingdom of Belgium | 1901.38  (3124.53, 1211.60) | 97.06  (159.03, 62.08) | 2124.13  (3343.26, 1361.88) | 114.35  (179.58, 73.54) | 0.45  (0.27, 0.63) |
| Kingdom of Bhutan | 50.13  (87.51, 28.71) | 24.76  (43.46, 13.95) | 78.43  (133.53, 45.82) | 38.72  (65.73, 22.68) | 1.66  (1.56, 1.76) |
| Kingdom of Cambodia | 1713.96  (2850.45, 994.82) | 54.84  (91.41, 31.67) | 3739.61  (6114.22, 2232.64) | 84.48  (137.99, 50.48) | 1.58  (1.53, 1.63) |
| Kingdom of Denmark | 964.43  (1564.96, 603.70) | 89.46  (144.75, 56.17) | 1103.49  (1775.48, 711.24) | 110.57  (177.38, 71.49) | 0.70  (0.60, 0.80) |
| Kingdom of Eswatini | 98.70  (167.98, 58.41) | 36.18  (61.84, 21.14) | 153.92  (262.12, 91.74) | 43.34  (73.82, 25.75) | 0.34  (0.15, 0.53) |
| Kingdom of Lesotho | 127.96  (211.40, 74.94) | 27.15  (45.14, 15.69) | 228.77  (384.32, 134.75) | 37.64  (63.31, 22.04) | 0.98  (0.88, 1.08) |
| Kingdom of Morocco | 4479.54  (7461.71, 2718.82) | 56.31  (93.95, 34.00) | 6461.37  (10764.15, 3950.65) | 70.46  (117.30, 43.13) | 0.79  (0.77, 0.81) |
| Kingdom of Norway | 885.71  (1472.62, 556.73) | 95.66  (158.70, 60.31) | 1030.66  (1712.12, 646.60) | 110.44  (183.02, 69.59) | 0.34  (0.23, 0.44) |
| Kingdom of Saudi Arabia | 2877.25  (4780.44, 1764.46) | 64.03  (106.78, 39.01) | 5957.80  (10136.75, 3696.97) | 85.28  (144.32, 53.44) | 1.02  (0.99, 1.04) |
| Kingdom of Spain | 8705.50  (14219.74, 5539.16) | 90.07  (147.05, 57.32) | 7655.64  (12482.81, 4847.41) | 112.26  (182.74, 71.36) | 0.59  (0.48, 0.71) |
| Kingdom of Sweden | 1284.26  (2093.25, 787.64) | 77.26  (125.52, 47.62) | 1613.49  (2633.69, 988.79) | 93.33  (152.16, 57.41) | 0.52  (0.31, 0.74) |
| Kingdom of Thailand | 13958.59  (23292.99, 8084.53) | 79.39  (132.40, 45.95) | 15265.87  (25899.33, 9392.43) | 131.58  (222.71, 81.30) | 1.75  (1.51, 1.98) |
| Kingdom of the Netherlands | 2769.98  (4509.43, 1754.75) | 84.70  (137.43, 53.86) | 3129.29  (5045.51, 1961.46) | 105.73  (170.12, 66.46) | 0.71  (0.66, 0.77) |
| Kingdom of Tonga | 23.06  (38.80, 14.49) | 71.21  (120.40, 44.38) | 28.14  (46.13, 17.65) | 92.83  (152.50, 58.05) | 0.64  (0.47, 0.81) |
| Kyrgyz Republic | 182.14  (315.50, 104.00) | 14.63  (25.45, 8.26) | 279.45  (483.41, 158.19) | 17.00  (29.28, 9.66) | 0.39  (0.32, 0.45) |
| Lao People's Democratic Republic | 784.43  (1315.39, 456.74) | 61.03  (102.68, 35.32) | 2040.19  (3361.87, 1203.85) | 99.21  (163.36, 58.55) | 1.84  (1.78, 1.90) |
| Lebanese Republic | 522.32  (893.91, 317.84) | 62.02  (106.16, 37.66) | 871.10  (1460.29, 541.41) | 77.88  (130.33, 48.72) | 0.77  (0.75, 0.80) |
| Malaysia | 5369.81  (9250.54, 3097.62) | 102.69  (176.99, 59.14) | 11515.01  (19716.07, 7111.93) | 149.17  (255.09, 92.27) | 1.48  (1.34, 1.62) |
| Mongolia | 94.33  (165.23, 53.03) | 14.25  (25.06, 7.89) | 128.60  (217.49, 73.57) | 18.57  (31.24, 10.79) | 0.96  (0.91, 1.00) |
| Montenegro | 10.67  (18.68, 6.02) | 6.88  (12.04, 3.89) | 10.01  (17.42, 5.58) | 8.57  (14.78, 4.87) | 0.86  (0.80, 0.92) |
| New Zealand | 1974.36  (3554.74, 1152.30) | 223.41  (402.26, 130.40) | 2186.94  (3719.35, 1312.10) | 233.96  (397.33, 140.65) | -0.04  (-0.24, 0.17) |
| North Macedonia | 30.40  (53.52, 16.11) | 6.07  (10.67, 3.22) | 30.48  (54.07, 16.22) | 8.03  (14.04, 4.46) | 0.96  (0.90, 1.02) |
| Northern Mariana Islands | 9.75  (16.56, 6.02) | 73.74  (124.69, 45.70) | 9.87  (16.46, 6.20) | 91.91  (153.33, 57.73) | 0.61  (0.48, 0.74) |
| Palestine | 357.88  (599.85, 216.98) | 55.69  (93.69, 33.50) | 1066.89  (1744.14, 661.00) | 68.27  (111.74, 42.15) | 0.66  (0.62, 0.70) |
| People's Democratic Republic of Algeria | 4444.53  (7386.98, 2688.86) | 55.33  (92.22, 33.25) | 6978.40  (11594.70, 4337.87) | 75.04  (124.47, 46.85) | 1.17  (1.12, 1.22) |
| People's Republic of Bangladesh | 6262.48  (10840.92, 3557.57) | 18.97  (32.96, 10.58) | 13224.00  (22367.14, 7759.07) | 28.13  (47.49, 16.52) | 1.51  (1.38, 1.64) |
| People's Republic of China | 134474.66  (229857.43, 77957.53) | 36.81  (62.65, 21.31) | 130403.31  (217207.79, 76467.10) | 60.42  (100.32, 35.84) | 1.62  (1.47, 1.77) |
| Plurinational State of Bolivia | 1367.04  (2266.94, 848.37) | 69.47  (115.51, 42.93) | 2769.44  (4465.47, 1803.86) | 88.26  (142.20, 57.51) | 0.77  (0.73, 0.81) |
| Portuguese Republic | 2093.50  (3423.36, 1306.93) | 82.28  (134.59, 51.35) | 1777.79  (2866.78, 1119.94) | 109.00  (175.41, 68.92) | 0.73  (0.57, 0.89) |
| Principality of Andorra | 10.91  (17.60, 7.02) | 95.44  (153.34, 61.66) | 14.13  (22.58, 8.96) | 116.35  (185.23, 74.21) | 0.66  (0.57, 0.76) |
| Principality of Monaco | 3.93  (6.37, 2.50) | 99.96  (160.57, 64.22) | 6.19  (9.95, 3.96) | 118.27  (189.87, 76.01) | 0.55  (0.48, 0.63) |
| Puerto Rico | 537.84  (867.26, 342.86) | 54.52  (87.99, 34.72) | 416.21  (665.24, 266.48) | 67.29  (107.22, 43.27) | 0.69  (0.61, 0.76) |
| Republic of Albania | 59.02  (103.73, 33.24) | 6.17  (10.88, 3.44) | 41.78  (73.64, 22.72) | 8.00  (13.95, 4.44) | 0.81  (0.74, 0.87) |
| Republic of Angola | 581.66  (1012.53, 332.63) | 19.76  (34.64, 11.10) | 3091.67  (5266.76, 1786.91) | 30.77  (52.79, 17.56) | 1.34  (1.22, 1.45) |
| Republic of Armenia | 119.12  (208.73, 67.62) | 14.08  (24.56, 8.03) | 100.46  (175.24, 56.48) | 19.46  (33.70, 11.25) | 1.19  (1.15, 1.23) |
| Republic of Austria | 1786.36  (2923.46, 1151.39) | 115.35  (187.96, 74.65) | 1647.95  (2704.86, 1059.98) | 124.51  (203.71, 80.49) | 0.08  (0.03, 0.14) |
| Republic of Azerbaijan | 289.59  (504.86, 164.47) | 14.26  (24.72, 8.10) | 407.94  (711.99, 231.47) | 19.82  (34.25, 11.47) | 1.28  (1.18, 1.37) |
| Republic of Belarus | 226.68  (397.38, 121.57) | 9.89  (17.29, 5.37) | 183.33  (325.64, 97.44) | 12.60  (22.26, 6.97) | 0.87  (0.82, 0.92) |
| Republic of Benin | 290.81  (503.55, 166.17) | 21.73  (37.66, 12.31) | 1452.31  (2482.46, 841.16) | 34.19  (58.68, 19.61) | 1.39  (1.19, 1.59) |
| Republic of Botswana | 132.23  (220.60, 78.56) | 29.67  (49.86, 17.39) | 273.37  (460.96, 161.49) | 41.87  (70.52, 24.78) | 1.27  (1.11, 1.43) |
| Republic of Bulgaria | 129.24  (230.08, 70.36) | 6.94  (12.34, 3.81) | 85.82  (149.31, 47.40) | 8.69  (14.98, 4.94) | 0.72  (0.68, 0.76) |
| Republic of Burundi | 331.26  (566.13, 188.36) | 21.14  (36.27, 11.88) | 915.41  (1543.75, 532.16) | 22.19  (37.73, 12.68) | 0.10  (0.05, 0.15) |
| Republic of Cabo Verde | 24.60  (42.37, 14.11) | 23.14  (40.01, 13.08) | 50.18  (84.73, 29.23) | 33.87  (57.06, 19.78) | 1.07  (0.91, 1.23) |
| Republic of Cameroon | 904.31  (1616.93, 518.40) | 29.06  (52.06, 16.47) | 3614.34  (6183.94, 2112.77) | 36.17  (62.16, 20.95) | 0.57  (0.49, 0.64) |
| Republic of Chad | 298.58  (505.22, 171.20) | 17.38  (29.60, 9.75) | 1295.62  (2158.84, 754.08) | 23.59  (39.72, 13.46) | 0.66  (0.48, 0.84) |
| Republic of Chile | 2196.99  (3714.14, 1264.87) | 56.82  (96.01, 32.69) | 3446.26  (5732.33, 1999.03) | 94.51  (156.75, 55.05) | 1.63  (1.33, 1.93) |
| Republic of Colombia | 5073.74  (7928.04, 3312.90) | 49.57  (77.49, 32.28) | 7339.30  (11490.92, 4746.18) | 63.40  (98.88, 41.16) | 0.68  (0.63, 0.73) |
| Republic of Costa Rica | 501.60  (782.53, 329.66) | 57.51  (89.68, 37.73) | 784.22  (1219.24, 514.14) | 73.85  (114.26, 48.68) | 0.70  (0.64, 0.76) |
| Republic of Croatia | 68.14  (119.94, 36.84) | 6.57  (11.50, 3.61) | 55.77  (99.22, 30.21) | 8.50  (14.93, 4.75) | 0.91  (0.83, 0.99) |
| Republic of Cuba | 1439.71  (2365.84, 899.63) | 43.16  (70.79, 26.94) | 1009.97  (1671.72, 627.96) | 54.67  (89.87, 34.34) | 0.78  (0.74, 0.82) |
| Republic of Cyprus | 139.65  (222.98, 87.58) | 79.03  (125.91, 49.68) | 215.49  (350.71, 136.24) | 110.23  (178.43, 70.38) | 1.19  (1.03, 1.35) |
| Republic of C么te d'Ivoire | 771.95  (1335.83, 439.20) | 21.86  (37.90, 12.29) | 2525.25  (4340.47, 1469.24) | 31.85  (54.91, 18.42) | 1.06  (0.85, 1.27) |
| Republic of Djibouti | 31.59  (53.42, 18.00) | 23.57  (40.18, 13.22) | 107.69  (185.43, 61.20) | 35.14  (60.42, 20.04) | 1.38  (1.28, 1.49) |
| Republic of Ecuador | 2745.27  (4568.98, 1746.92) | 84.12  (140.24, 53.34) | 4825.26  (7695.62, 3288.04) | 105.59  (168.19, 72.04) | 0.62  (0.36, 0.88) |
| Republic of El Salvador | 881.97  (1371.57, 572.78) | 49.49  (77.35, 31.91) | 1129.98  (1726.95, 745.45) | 67.42  (102.74, 44.59) | 0.85  (0.73, 0.97) |
| Republic of Equatorial Guinea | 27.57  (46.85, 15.95) | 21.85  (37.29, 12.46) | 184.89  (315.92, 110.96) | 41.30  (70.74, 24.64) | 2.22  (1.89, 2.55) |
| Republic of Estonia | 34.75  (62.31, 18.45) | 10.41  (18.64, 5.61) | 27.79  (49.61, 14.86) | 13.72  (24.45, 7.53) | 1.08  (1.01, 1.15) |
| Republic of Fiji | 132.77  (223.65, 80.10) | 59.13  (99.66, 35.58) | 191.97  (315.13, 118.54) | 84.16  (138.16, 51.97) | 1.07  (0.97, 1.18) |
| Republic of Finland | 808.68  (1299.00, 508.99) | 90.13  (144.20, 57.04) | 981.37  (1574.39, 624.50) | 111.03  (177.86, 70.83) | 0.67  (0.61, 0.73) |
| Republic of Ghana | 1003.92  (1725.65, 576.84) | 22.78  (39.29, 12.94) | 3090.21  (5279.85, 1770.35) | 30.60  (52.26, 17.48) | 0.72  (0.54, 0.91) |
| Republic of Guatemala | 1120.92  (1760.24, 730.39) | 43.86  (69.44, 28.29) | 2983.08  (4567.21, 1956.05) | 59.40  (90.96, 38.87) | 0.83  (0.73, 0.93) |
| Republic of Guinea | 323.08  (547.71, 184.48) | 20.40  (34.70, 11.53) | 1172.43  (1974.01, 681.64) | 27.92  (47.24, 16.06) | 0.86  (0.75, 0.96) |
| Republic of Guinea-Bissau | 62.41  (106.95, 35.93) | 20.51  (35.40, 11.62) | 180.08  (307.79, 103.85) | 27.99  (48.03, 15.99) | 0.78  (0.58, 0.98) |
| Republic of Guyana | 95.80  (157.60, 58.33) | 36.89  (60.85, 22.28) | 100.02  (163.12, 62.33) | 49.78  (80.85, 31.10) | 0.95  (0.87, 1.03) |
| Republic of Haiti | 571.86  (973.76, 343.45) | 30.06  (51.41, 17.90) | 1352.35  (2187.16, 837.16) | 36.26  (58.69, 22.41) | 0.69  (0.66, 0.73) |
| Republic of Honduras | 624.48  (976.75, 403.61) | 42.18  (66.38, 27.00) | 1911.82  (2989.79, 1254.18) | 59.99  (93.81, 39.29) | 1.06  (0.95, 1.18) |
| Republic of Iceland | 59.99  (98.61, 38.30) | 97.47  (160.03, 62.30) | 76.63  (124.61, 49.32) | 120.09  (194.92, 77.50) | 0.74  (0.67, 0.82) |
| Republic of India | 68368.82  (112374.41, 41181.23) | 29.31  (48.25, 17.54) | 180499.99  (296810.40, 107450.51) | 46.94  (77.04, 27.96) | 1.78  (1.66, 1.91) |
| Republic of Indonesia | 39723.49  (66516.94, 23098.38) | 68.24  (114.38, 39.54) | 75391.80  (123076.45, 45541.73) | 112.78  (183.76, 68.28) | 1.94  (1.84, 2.03) |
| Republic of Iraq | 3603.70  (6188.90, 2213.76) | 62.36  (107.49, 38.01) | 8456.96  (14019.97, 5297.71) | 70.58  (117.13, 44.09) | 0.56  (0.46, 0.65) |
| Republic of Italy | 28353.74  (41835.32, 19890.05) | 220.25  (324.59, 154.66) | 16867.25  (24247.12, 11942.64) | 201.75  (289.41, 143.26) | -0.56  (-0.70, -0.42) |
| Republic of Kazakhstan | 655.82  (1125.17, 368.50) | 15.49  (26.59, 8.69) | 798.79  (1385.24, 458.07) | 20.32  (35.18, 11.77) | 0.91  (0.90, 0.93) |
| Republic of Kenya | 2149.55  (3670.82, 1245.65) | 29.27  (50.21, 16.70) | 5640.92  (9640.31, 3257.31) | 33.67  (57.74, 19.25) | 0.40  (0.32, 0.47) |
| Republic of Kiribati | 10.99  (18.23, 6.67) | 52.50  (86.84, 31.83) | 24.69  (40.08, 15.58) | 72.29  (117.40, 45.56) | 0.95  (0.77, 1.14) |
| Republic of Korea | 15889.09  (25202.02, 9110.70) | 119.21  (189.04, 68.34) | 12493.66  (19877.93, 7052.22) | 185.19  (293.32, 105.04) | 1.12  (0.75, 1.48) |
| Republic of Latvia | 58.18  (103.03, 31.14) | 10.35  (18.26, 5.63) | 36.65  (64.72, 19.51) | 12.97  (22.81, 7.12) | 0.84  (0.79, 0.88) |
| Republic of Liberia | 156.89  (267.66, 90.15) | 22.48  (38.47, 12.75) | 522.03  (886.24, 302.68) | 30.02  (51.23, 17.23) | 1.02  (0.93, 1.11) |
| Republic of Lithuania | 81.64  (146.24, 44.23) | 9.67  (17.27, 5.29) | 52.92  (94.55, 27.79) | 12.61  (22.28, 6.85) | 0.99  (0.94, 1.04) |
| Republic of Madagascar | 813.80  (1424.49, 470.17) | 22.53  (39.67, 12.81) | 2335.76  (3957.33, 1341.92) | 25.03  (42.65, 14.17) | 0.40  (0.36, 0.43) |
| Republic of Malawi | 838.06  (1491.58, 478.55) | 27.94  (49.75, 15.79) | 2220.82  (3848.93, 1300.94) | 31.14  (54.35, 17.99) | 0.41  (0.33, 0.49) |
| Republic of Maldives | 43.68  (74.02, 25.56) | 63.89  (108.54, 37.16) | 106.81  (180.58, 63.85) | 125.64  (212.02, 75.43) | 2.86  (2.61, 3.11) |
| Republic of Mali | 430.73  (727.48, 242.77) | 18.28  (30.96, 10.15) | 1955.23  (3261.04, 1143.34) | 25.89  (43.44, 14.92) | 0.94  (0.79, 1.10) |
| Republic of Malta | 71.62  (116.93, 45.55) | 89.07  (145.40, 56.73) | 69.14  (112.65, 44.26) | 117.29  (190.29, 75.57) | 0.87  (0.73, 1.01) |
| Republic of Mauritius | 302.33  (523.40, 175.50) | 101.42  (175.27, 58.93) | 370.68  (633.91, 225.15) | 139.27  (237.87, 84.76) | 1.28  (1.17, 1.39) |
| Republic of Moldova | 91.92  (164.71, 48.10) | 8.78  (15.76, 4.63) | 69.49  (119.65, 36.46) | 11.83  (20.13, 6.47) | 1.15  (1.04, 1.26) |
| Republic of Mozambique | 903.75  (1537.18, 525.24) | 22.10  (37.95, 12.63) | 3134.86  (5421.20, 1801.40) | 30.08  (52.25, 17.08) | 1.01  (0.95, 1.07) |
| Republic of Namibia | 133.48  (224.34, 78.18) | 28.61  (48.43, 16.50) | 266.53  (449.84, 157.12) | 36.30  (61.32, 21.33) | 0.72  (0.62, 0.82) |
| Republic of Nauru | 1.84  (3.04, 1.12) | 63.43  (104.75, 38.40) | 2.87  (4.78, 1.79) | 86.56  (144.39, 53.94) | 0.91  (0.85, 0.96) |
| Republic of Nicaragua | 601.68  (954.78, 392.09) | 47.05  (75.08, 30.39) | 1152.83  (1755.07, 757.07) | 63.70  (96.84, 41.86) | 0.89  (0.80, 0.97) |
| Republic of Niue | 0.39  (0.66, 0.24) | 67.80  (115.88, 41.44) | 0.36  (0.60, 0.23) | 95.33  (158.42, 60.59) | 1.06  (0.92, 1.19) |
| Republic of Palau | 3.24  (5.40, 1.99) | 70.33  (117.36, 43.05) | 3.18  (5.35, 2.00) | 95.35  (160.62, 59.85) | 0.89  (0.76, 1.02) |
| Republic of Panama | 324.41  (512.39, 210.31) | 43.86  (69.44, 28.29) | 673.28  (1059.05, 437.04) | 65.13  (102.21, 42.34) | 1.13  (1.06, 1.20) |
| Republic of Paraguay | 185.47  (321.24, 105.44) | 16.18  (28.11, 9.09) | 452.19  (785.12, 265.57) | 23.29  (40.38, 13.68) | 1.33  (1.29, 1.38) |
| Republic of Peru | 5111.90  (8570.60, 3206.13) | 73.35  (123.16, 45.82) | 7849.78  (12554.81, 5013.59) | 93.18  (148.57, 59.70) | 0.78  (0.75, 0.82) |
| Republic of Poland | 939.76  (1679.80, 521.11) | 11.01  (19.77, 6.14) | 563.53  (923.30, 334.61) | 9.66  (15.54, 5.95) | -0.91  (-1.16, -0.66) |
| Republic of Rwanda | 565.58  (969.99, 325.93) | 26.03  (44.97, 14.77) | 1319.30  (2233.96, 757.13) | 30.64  (52.06, 17.43) | 0.66  (0.62, 0.71) |
| Republic of San Marino | 5.45  (8.87, 3.46) | 96.30  (156.29, 61.28) | 6.15  (9.87, 3.90) | 115.08  (184.35, 73.06) | 0.56  (0.48, 0.64) |
| Republic of Senegal | 584.87  (1019.94, 333.03) | 24.97  (43.81, 13.98) | 1439.04  (2488.31, 827.66) | 29.61  (51.39, 16.87) | 0.36  (0.27, 0.45) |
| Republic of Serbia | 135.23  (247.70, 73.95) | 6.24  (11.40, 3.43) | 131.24  (232.93, 70.97) | 8.06  (14.21, 4.46) | 0.94  (0.89, 1.00) |
| Republic of Seychelles | 23.47  (41.19, 13.58) | 104.97  (184.23, 60.64) | 27.74  (46.01, 16.95) | 132.81  (220.14, 81.26) | 0.85  (0.79, 0.91) |
| Republic of Sierra Leone | 239.00  (403.27, 138.46) | 20.20  (34.06, 11.62) | 867.89  (1501.42, 506.41) | 29.98  (51.95, 17.33) | 1.15  (1.04, 1.25) |
| Republic of Singapore | 1037.50  (1673.53, 596.54) | 136.70  (219.57, 78.85) | 1428.25  (2284.29, 794.48) | 219.56  (349.57, 122.83) | 1.58  (1.43, 1.73) |
| Republic of Slovenia | 29.30  (51.84, 16.10) | 6.69  (11.75, 3.73) | 25.51  (45.06, 13.89) | 8.81  (15.37, 4.96) | 0.93  (0.85, 1.02) |
| Republic of South Africa | 4690.77  (7873.48, 2729.31) | 39.36  (66.20, 22.75) | 6883.10  (11724.34, 4014.75) | 48.64  (82.77, 28.46) | 0.71  (0.66, 0.76) |
| Republic of South Sudan | 429.52  (731.65, 244.55) | 24.10  (41.31, 13.50) | 837.48  (1417.46, 480.14) | 26.10  (44.62, 14.72) | 0.15  (0.06, 0.23) |
| Republic of Sudan | 2539.82  (4268.03, 1494.48) | 40.86  (69.00, 23.81) | 8809.22  (14613.53, 5463.06) | 64.40  (107.04, 39.77) | 1.59  (1.47, 1.72) |
| Republic of Suriname | 46.51  (75.96, 28.68) | 39.94  (65.29, 24.52) | 70.49  (117.49, 43.48) | 52.00  (86.52, 32.15) | 0.80  (0.77, 0.84) |
| Republic of Tajikistan | 196.57  (339.53, 112.41) | 12.52  (21.75, 7.03) | 390.79  (671.19, 219.71) | 15.27  (26.14, 8.61) | 0.73  (0.67, 0.79) |
| Republic of the Congo | 198.29  (345.29, 114.48) | 25.34  (44.37, 14.39) | 535.00  (912.26, 312.90) | 32.53  (55.67, 18.89) | 0.69  (0.58, 0.80) |
| Republic of the Gambia | 64.74  (110.17, 36.61) | 21.94  (37.48, 12.25) | 239.39  (403.27, 138.91) | 29.75  (50.40, 17.07) | 0.79  (0.66, 0.92) |
| Republic of the Marshall Islands | 6.40  (10.42, 3.89) | 46.63  (76.41, 28.02) | 11.77  (19.53, 7.31) | 71.66  (119.04, 44.42) | 1.23  (1.08, 1.38) |
| Republic of the Niger | 432.96  (732.63, 246.52) | 18.81  (32.06, 10.51) | 1881.22  (3150.14, 1106.07) | 23.47  (39.77, 13.51) | 0.68  (0.57, 0.79) |
| Republic of the Philippines | 14769.15  (24594.77, 8711.06) | 74.04  (123.60, 43.47) | 34822.19  (57318.89, 20771.16) | 111.16  (182.90, 66.27) | 1.62  (1.53, 1.71) |
| Republic of the Union of Myanmar | 7391.41  (12400.69, 4269.66) | 57.06  (95.88, 32.85) | 15547.05  (25477.55, 9422.54) | 101.12  (165.67, 61.28) | 2.34  (2.20, 2.48) |
| Republic of Trinidad and Tobago | 134.07  (221.75, 82.42) | 40.47  (66.97, 24.84) | 148.61  (243.13, 92.39) | 54.49  (89.03, 34.06) | 1.05  (0.94, 1.16) |
| Republic of Tunisia | 1387.83  (2262.50, 861.46) | 54.32  (88.70, 33.55) | 1727.92  (2900.65, 1074.78) | 71.81  (120.34, 44.91) | 1.03  (0.99, 1.07) |
| Republic of Turkey | 9297.59  (15064.39, 5657.65) | 52.13  (84.72, 31.53) | 12484.80  (20717.05, 7614.36) | 68.55  (113.39, 42.01) | 1.01  (0.96, 1.06) |
| Republic of Uganda | 1355.59  (2343.79, 776.67) | 24.78  (43.09, 13.95) | 4226.63  (7119.87, 2441.13) | 29.21  (49.53, 16.62) | 0.56  (0.54, 0.58) |
| Republic of Uzbekistan | 925.76  (1576.79, 527.04) | 15.10  (25.82, 8.50) | 1487.82  (2544.38, 846.22) | 19.49  (33.10, 11.23) | 0.88  (0.77, 0.99) |
| Republic of Vanuatu | 23.06  (37.60, 14.06) | 51.83  (84.69, 31.40) | 64.47  (105.88, 40.50) | 72.21  (118.64, 45.26) | 1.03  (0.98, 1.08) |
| Republic of Yemen | 1529.33  (2507.36, 924.63) | 41.83  (69.00, 25.05) | 5267.92  (8627.45, 3283.18) | 49.86  (82.15, 30.84) | 0.86  (0.76, 0.97) |
| Republic of Zambia | 841.62  (1467.82, 481.58) | 30.50  (53.50, 17.18) | 2301.38  (3950.08, 1312.84) | 35.60  (61.34, 20.13) | 0.43  (0.39, 0.46) |
| Republic of Zimbabwe | 1107.08  (1871.15, 662.81) | 31.73  (54.01, 18.71) | 1693.84  (2861.87, 1023.48) | 33.89  (57.46, 20.30) | -0.05  (-0.19, 0.09) |
| Romania | 352.09  (644.78, 193.54) | 6.24  (11.40, 3.43) | 261.08  (457.43, 141.51) | 8.37  (14.54, 4.64) | 1.03  (0.98, 1.08) |
| Russian Federation | 3975.44  (6809.49, 2128.62) | 11.98  (20.60, 6.49) | 3674.96  (6281.22, 1964.51) | 14.66  (25.12, 8.03) | 0.75  (0.72, 0.78) |
| Saint Kitts and Nevis | 5.50  (9.01, 3.37) | 44.21  (72.59, 26.92) | 7.29  (11.79, 4.63) | 57.04  (91.88, 36.44) | 0.76  (0.70, 0.82) |
| Saint Lucia | 17.52  (29.45, 10.95) | 39.07  (65.89, 24.21) | 17.92  (29.77, 10.93) | 49.50  (81.81, 30.46) | 0.60  (0.49, 0.70) |
| Saint Vincent and the Grenadines | 13.03  (21.16, 8.05) | 36.03  (58.82, 22.02) | 12.87  (20.95, 7.96) | 50.39  (81.83, 31.24) | 1.11  (1.02, 1.20) |
| Slovak Republic | 80.84  (143.65, 44.22) | 6.39  (11.37, 3.50) | 71.44  (125.74, 38.60) | 8.41  (14.59, 4.72) | 0.93  (0.89, 0.97) |
| Socialist Republic of Viet Nam | 11516.90  (18960.40, 6763.89) | 54.15  (89.31, 31.65) | 19036.87  (30571.55, 11302.11) | 94.98  (152.26, 56.60) | 2.36  (2.19, 2.53) |
| Solomon Islands | 50.84  (82.72, 30.98) | 46.38  (75.95, 27.96) | 135.76  (225.69, 83.71) | 67.47  (112.33, 41.48) | 1.04  (0.84, 1.24) |
| State of Eritrea | 194.96  (330.14, 112.03) | 19.15  (32.65, 10.84) | 501.28  (844.52, 288.13) | 25.82  (43.61, 14.73) | 0.95  (0.81, 1.09) |
| State of Israel | 1140.33  (1827.11, 726.63) | 83.57  (134.19, 53.15) | 2314.97  (3774.44, 1469.17) | 106.66  (173.92, 67.70) | 0.73  (0.64, 0.82) |
| State of Kuwait | 301.69  (511.61, 187.05) | 74.29  (125.36, 46.23) | 659.17  (1100.13, 411.33) | 87.10  (144.48, 55.23) | 0.70  (0.65, 0.75) |
| State of Libya | 985.71  (1661.21, 611.32) | 68.52  (116.14, 42.10) | 1320.33  (2202.81, 820.61) | 75.31  (125.50, 46.93) | 0.44  (0.40, 0.49) |
| State of Qatar | 55.62  (95.17, 33.98) | 74.55  (127.68, 45.60) | 271.78  (456.05, 167.87) | 86.38  (144.52, 54.06) | 0.51  (0.49, 0.54) |
| Sultanate of Oman | 232.25  (386.23, 140.87) | 53.62  (89.52, 32.31) | 640.72  (1048.42, 396.80) | 80.03  (130.68, 49.81) | 1.42  (1.34, 1.49) |
| Swiss Confederation | 1144.25  (1901.71, 716.33) | 92.29  (152.55, 58.14) | 1323.19  (2191.04, 826.63) | 105.19  (173.57, 66.15) | 0.48  (0.44, 0.51) |
| Syrian Arab Republic | 2286.42  (3761.65, 1400.64) | 56.05  (92.69, 34.00) | 3688.81  (6162.25, 2269.48) | 70.69  (118.53, 43.22) | 0.83  (0.76, 0.90) |
| Taiwan (Province of China) | 3097.44  (5437.80, 1830.02) | 58.42  (102.38, 34.60) | 2832.85  (4796.45, 1751.08) | 87.39  (146.99, 54.74) | 1.46  (1.39, 1.53) |
| Togolese Republic | 232.92  (402.66, 133.86) | 20.99  (36.44, 11.87) | 706.81  (1204.57, 407.98) | 28.81  (49.31, 16.51) | 0.81  (0.68, 0.93) |
| Tokelau | 0.26  (0.42, 0.16) | 58.93  (96.69, 35.30) | 0.30  (0.50, 0.19) | 88.65  (147.66, 55.48) | 1.24  (1.12, 1.36) |
| Turkmenistan | 159.80  (276.16, 90.46) | 14.71  (25.49, 8.24) | 245.43  (425.73, 139.60) | 19.64  (34.03, 11.18) | 0.95  (0.92, 0.98) |
| Tuvalu | 1.33  (2.18, 0.81) | 55.86  (91.30, 34.10) | 2.71  (4.53, 1.68) | 82.46  (137.76, 50.90) | 1.09  (0.95, 1.24) |
| Ukraine | 1316.00  (2233.85, 703.50) | 11.49  (19.53, 6.21) | 958.73  (1671.60, 501.81) | 13.32  (23.30, 7.22) | 0.62  (0.57, 0.67) |
| Union of the Comoros | 40.82  (70.75, 23.36) | 27.94  (48.71, 15.75) | 69.04  (120.44, 40.27) | 31.96  (55.80, 18.56) | 0.25  (0.13, 0.38) |
| United Arab Emirates | 207.58  (354.93, 127.94) | 64.10  (109.20, 39.67) | 846.15  (1425.57, 526.50) | 80.37  (135.36, 50.51) | 0.75  (0.66, 0.84) |
| United Kingdom of Great Britain and Northern Ireland | 11858.62  (19394.09, 7494.58) | 102.71  (167.31, 65.21) | 14584.38  (23782.72, 9278.50) | 127.51  (207.41, 81.45) | 0.51  (0.41, 0.62) |
| United Mexican States | 28083.43  (41872.79, 19134.55) | 94.53  (141.47, 64.08) | 30941.73  (45844.25, 21339.98) | 93.93  (138.96, 64.87) | -0.49  (-0.71, -0.27) |
| United Republic of Tanzania | 2292.05  (4002.19, 1302.88) | 27.33  (47.94, 15.29) | 6142.04  (10531.80, 3563.12) | 32.12  (55.28, 18.45) | 0.52  (0.44, 0.59) |
| United States of America | 69376.96  (121207.36, 39941.08) | 130.48  (227.60, 75.32) | 126400.31  (198907.51, 74092.10) | 197.42  (310.40, 115.84) | -0.33  (-1.03, 0.38) |
| United States Virgin Islands | 15.40  (24.99, 9.78) | 53.24  (86.76, 33.73) | 9.38  (15.25, 6.05) | 65.29  (106.13, 42.21) | 0.64  (0.55, 0.72) |

Abbreviations: ASIR, age standardized incidence rate; EAPC, estimated annual percentage change; SDI, socio-demographic index; UI, uncertainty interval; CI, confidence interval.
