# Supplementary material for: Global burden of polycystic ovary syndrome in women of reproductive age, 1990–2021: Analysis of the global burden of disease study 2021 with projections to 2050
Source: PLoS One. 2025 Oct 7;20(10):e0333000. doi: 10.1371/journal.pone.0333000 (PMC12503323; doi:10.1371/journal.pone.0333000)
Supplement: S4 Table — (DOCX) [file pone.0333000.s014.docx]

Table 5. Prevalence of PCOS among women of reproductive age between 1990 and 2021 at the national level.

| Nations | 1990 | | 2021 | | 1990-2021 EAPC |
| --- | --- | --- | --- | --- | --- |
|  | All-age cases | ASIR per 100,000 population | All-age cases | ASIR per 100,000 population |  |
|  | n*10^3^ (95% UI) | n (95% UI) | n*10^3^ (95% UI) | n (95% UI) | n (95% CI) |
| American Samoa | 464.87  (658.42, 317.43) | 3845.00  (5442.02, 2628.20) | 614.73  (891.98, 420.73) | 5315.55  (7714.10, 3642.68) | 0.90  (0.72, 1.08) |
| Antigua and Barbuda | 368.59  (528.31, 246.32) | 2251.21  (3229.05, 1505.68) | 722.66  (1030.60, 489.77) | 2997.85  (4278.36, 2026.30) | 0.81  (0.75, 0.86) |
| Arab Republic of Egypt | 456387.67  (653487.28, 309106.34) | 3480.04  (4978.93, 2360.65) | 1146698.04  (1639791.28, 791187.79) | 4406.99  (6300.09, 3040.68) | 0.63  (0.56, 0.69) |
| Argentine Republic | 178995.02  (259070.17, 123119.41) | 2239.19  (3239.02, 1540.58) | 411902.79  (592358.35, 287030.55) | 3450.24  (4962.20, 2404.42) | 1.35  (1.17, 1.52) |
| Australia | 328293.95  (439413.83, 236836.21) | 7319.76  (9797.76, 5280.24) | 534957.32  (753153.92, 374256.41) | 8818.88  (12413.72, 6166.58) | 0.38  (0.25, 0.50) |
| Barbados | 1945.72  (2787.13, 1315.93) | 2819.15  (4038.74, 1906.84) | 2346.60  (3342.41, 1592.72) | 3318.26  (4723.37, 2246.66) | 0.47  (0.44, 0.51) |
| Belize | 941.72  (1340.70, 630.98) | 2233.04  (3175.64, 1503.63) | 3898.30  (5533.23, 2649.57) | 3225.15  (4575.62, 2195.05) | 0.97  (0.70, 1.24) |
| Bermuda | 588.50  (848.46, 386.43) | 3415.02  (4928.77, 2237.51) | 507.35  (736.74, 343.31) | 3800.59  (5529.51, 2561.92) | 0.24  (0.18, 0.30) |
| Bolivarian Republic of Venezuela | 185085.42  (270380.84, 123083.07) | 3796.62  (5540.50, 2525.37) | 325887.82  (474929.83, 219316.52) | 4777.65  (6961.74, 3214.71) | 0.67  (0.60, 0.74) |
| Bosnia and Herzegovina | 2742.84  (4343.66, 1713.77) | 234.51  (371.57, 146.49) | 2673.46  (4014.51, 1737.43) | 364.77  (551.34, 234.91) | 1.59  (1.36, 1.81) |
| Brunei Darussalam | 3192.77  (4631.89, 2155.36) | 4702.13  (6840.31, 3177.68) | 9324.79  (13291.46, 6411.06) | 7355.42  (10478.12, 5060.01) | 1.51  (1.35, 1.67) |
| Burkina Faso | 17181.60  (24793.02, 11550.14) | 828.78  (1188.84, 560.33) | 72544.46  (104847.31, 49038.26) | 1324.59  (1907.54, 898.02) | 1.29  (1.08, 1.50) |
| Canada | 206437.50  (299392.31, 142997.16) | 2766.41  (4010.60, 1914.61) | 295926.16  (431065.52, 203982.47) | 3546.13  (5163.66, 2445.90) | 0.72  (0.62, 0.83) |
| Central African Republic | 6477.69  (9542.78, 4414.81) | 1018.78  (1493.01, 696.32) | 15129.54  (21698.70, 10216.25) | 1104.23  (1578.85, 749.77) | 0.05  (-0.08, 0.18) |
| Commonwealth of Dominica | 380.60  (554.26, 255.67) | 2236.60  (3247.45, 1506.64) | 503.59  (729.87, 335.69) | 3067.91  (4446.14, 2045.04) | 0.86  (0.74, 0.98) |
| Commonwealth of the Bahamas | 2087.68  (3004.63, 1401.60) | 2857.94  (4106.50, 1922.98) | 3660.54  (5311.41, 2448.98) | 3409.45  (4948.02, 2280.10) | 0.52  (0.44, 0.59) |
| Cook Islands | 181.65  (264.75, 123.06) | 3966.11  (5781.82, 2691.70) | 252.48  (363.87, 171.33) | 5892.50  (8493.04, 3998.00) | 1.12  (0.99, 1.26) |
| Czech Republic | 7927.30  (11925.72, 4993.65) | 308.40  (464.86, 193.70) | 9041.63  (13484.93, 5925.91) | 396.05  (595.81, 256.97) | 0.78  (0.73, 0.84) |
| Democratic People's Republic of Korea | 94970.09  (138305.71, 66358.87) | 1678.50  (2442.00, 1174.32) | 124320.18  (177310.70, 85107.59) | 1882.44  (2684.85, 1287.90) | 0.32  (0.23, 0.41) |
| Democratic Republic of Sao Tome and Principe | 246.58  (359.10, 165.27) | 974.33  (1411.72, 658.13) | 808.75  (1171.91, 546.05) | 1447.41  (2092.55, 979.79) | 1.05  (0.84, 1.25) |
| Democratic Republic of the Congo | 70428.54  (103034.70, 46861.20) | 835.96  (1216.90, 559.61) | 257725.88  (372780.07, 173877.84) | 1218.93  (1755.55, 825.61) | 1.19  (1.02, 1.35) |
| Democratic Republic of Timor-Leste | 4456.54  (6339.83, 3048.21) | 2383.18  (3390.18, 1630.63) | 13500.70  (19557.68, 9342.16) | 3938.39  (5700.70, 2731.85) | 1.96  (1.85, 2.07) |
| Democratic Socialist Republic of Sri Lanka | 167583.62  (243116.01, 116928.50) | 3642.38  (5282.93, 2539.74) | 341198.33  (483393.75, 235167.57) | 6055.35  (8579.01, 4172.08) | 1.86  (1.64, 2.08) |
| Dominican Republic | 36972.20  (53500.63, 24747.61) | 1954.98  (2829.04, 1311.74) | 89880.41  (131138.25, 59902.83) | 3092.81  (4513.28, 2061.25) | 1.55  (1.46, 1.64) |
| Eastern Republic of Uruguay | 17184.27  (24947.09, 11839.08) | 2291.62  (3326.80, 1578.96) | 31556.17  (46027.25, 21952.68) | 3781.93  (5514.93, 2630.86) | 1.61  (1.42, 1.81) |
| Federal Democratic Republic of Ethiopia | 82940.95  (120126.35, 56763.79) | 750.56  (1076.14, 519.65) | 312483.86  (450602.28, 214893.24) | 1142.60  (1639.21, 792.13) | 1.56  (1.50, 1.63) |
| Federal Democratic Republic of Nepal | 33466.57  (48327.89, 22670.95) | 739.07  (1060.79, 502.78) | 114523.66  (162863.32, 76732.34) | 1255.47  (1783.34, 841.66) | 1.81  (1.76, 1.86) |
| Federal Republic of Germany | 922889.51  (1277556.12, 647377.48) | 4697.83  (6516.21, 3283.99) | 1011058.53  (1419299.14, 704802.99) | 5882.79  (8269.39, 4096.09) | 0.65  (0.59, 0.70) |
| Federal Republic of Nigeria | 205802.10  (298266.45, 139277.35) | 1038.18  (1490.70, 711.26) | 800539.89  (1147706.87, 545138.56) | 1418.26  (2023.71, 972.87) | 0.74  (0.56, 0.91) |
| Federal Republic of Somalia | 15363.72  (22214.29, 10394.38) | 914.77  (1316.00, 622.82) | 51130.57  (75369.98, 34554.96) | 1068.11  (1564.60, 726.60) | 0.59  (0.55, 0.64) |
| Federated States of Micronesia | 661.64  (946.99, 453.71) | 2867.47  (4097.58, 1974.58) | 1079.99  (1542.49, 741.97) | 4149.01  (5923.55, 2852.97) | 0.97  (0.76, 1.17) |
| Federative Republic of Brazil | 411775.80  (600927.95, 277017.82) | 1057.06  (1539.81, 714.19) | 672524.35  (959886.41, 459121.32) | 1140.88  (1631.48, 776.65) | -0.21  (-0.39, -0.03) |
| French Republic | 700299.65  (1002357.23, 477424.00) | 4816.24  (6890.08, 3283.49) | 858878.44  (1225428.10, 585958.84) | 6058.46  (8652.34, 4126.39) | 0.70  (0.66, 0.74) |
| Gabonese Republic | 2705.47  (3911.21, 1809.75) | 1233.86  (1773.23, 829.84) | 9094.44  (13508.87, 6010.14) | 1861.45  (2759.73, 1233.45) | 1.18  (1.03, 1.32) |
| Georgia | 10819.53  (15789.85, 7092.83) | 783.06  (1143.39, 512.81) | 10833.35  (15266.11, 7363.29) | 1379.37  (1946.97, 935.32) | 2.31  (2.06, 2.56) |
| Grand Duchy of Luxembourg | 5303.22  (7610.84, 3591.91) | 5382.48  (7729.42, 3645.47) | 10819.68  (15358.11, 7454.33) | 6888.97  (9785.91, 4742.87) | 0.79  (0.74, 0.85) |
| Greenland | 358.48  (508.64, 248.17) | 2364.63  (3356.30, 1635.33) | 404.27  (578.48, 280.67) | 3139.38  (4494.44, 2180.13) | 0.93  (0.84, 1.02) |
| Grenada | 376.17  (544.40, 250.04) | 1927.57  (2788.91, 1286.86) | 707.67  (1017.25, 473.55) | 2764.78  (3975.98, 1849.60) | 1.00  (0.87, 1.14) |
| Guam | 1497.88  (2154.90, 1013.98) | 4248.25  (6117.49, 2876.74) | 2143.44  (3041.13, 1466.80) | 5967.62  (8467.62, 4081.27) | 1.11  (1.02, 1.20) |
| Hashemite Kingdom of Jordan | 26160.23  (37438.86, 17859.42) | 3135.19  (4478.29, 2144.74) | 122992.81  (174070.61, 84627.15) | 3972.24  (5619.07, 2737.61) | 0.87  (0.81, 0.93) |
| Hellenic Republic | 138879.62  (197635.72, 94751.34) | 5503.14  (7835.36, 3752.86) | 149093.79  (212197.31, 102311.22) | 6886.58  (9802.15, 4721.70) | 0.52  (0.34, 0.70) |
| Hungary | 8409.11  (12573.84, 5345.18) | 332.13  (497.99, 210.41) | 8738.48  (13172.71, 5706.65) | 410.28  (622.51, 266.44) | 0.68  (0.60, 0.75) |
| Independent State of Papua New Guinea | 20455.96  (29147.78, 13927.97) | 2122.84  (3021.51, 1448.64) | 82232.33  (118393.58, 56189.20) | 3128.83  (4505.22, 2137.81) | 1.00  (0.83, 1.17) |
| Independent State of Samoa | 1255.98  (1836.30, 847.63) | 3434.84  (5021.97, 2326.76) | 2247.02  (3221.37, 1533.96) | 4636.23  (6647.75, 3169.76) | 0.86  (0.73, 0.99) |
| Ireland | 46608.38  (67103.67, 31975.00) | 5305.13  (7634.07, 3641.37) | 76762.69  (109281.75, 53453.97) | 6587.16  (9384.99, 4579.85) | 0.66  (0.56, 0.75) |
| Islamic Republic of Afghanistan | 37143.50  (53260.18, 25190.39) | 1725.83  (2458.51, 1176.93) | 177431.16  (253171.99, 120775.61) | 2480.11  (3536.79, 1691.03) | 1.68  (1.40, 1.97) |
| Islamic Republic of Iran | 403579.66  (574128.26, 278018.51) | 3197.28  (4544.38, 2211.97) | 990122.58  (1417796.73, 687111.21) | 4240.11  (6070.59, 2933.54) | 1.37  (1.01, 1.73) |
| Islamic Republic of Mauritania | 5345.03  (7906.63, 3631.03) | 1141.92  (1681.57, 780.10) | 17217.22  (24859.90, 11635.73) | 1609.40  (2313.76, 1092.70) | 0.76  (0.57, 0.96) |
| Islamic Republic of Pakistan | 293225.84  (422787.71, 200103.72) | 1259.71  (1805.16, 865.90) | 889148.93  (1272104.87, 604634.70) | 1468.96  (2097.27, 1000.91) | 0.54  (0.39, 0.69) |
| Jamaica | 13004.38  (18586.97, 8717.10) | 2172.46  (3100.77, 1461.11) | 22663.68  (32488.60, 15250.76) | 2922.68  (4190.79, 1965.20) | 0.97  (0.89, 1.04) |
| Japan | 3663284.37  (5185049.06, 2565544.61) | 11408.86  (16130.47, 7992.70) | 3065760.08  (4329767.53, 2156165.45) | 12234.24  (17244.66, 8601.85) | 0.21  (0.16, 0.26) |
| Kingdom of Bahrain | 4700.93  (6771.83, 3190.23) | 4017.43  (5781.19, 2727.48) | 14957.24  (21464.18, 10278.67) | 4579.65  (6574.31, 3144.31) | 0.40  (0.35, 0.46) |
| Kingdom of Belgium | 140145.71  (201055.71, 95779.32) | 5706.21  (8191.26, 3896.55) | 168222.97  (236183.84, 117429.07) | 6761.69  (9503.07, 4714.53) | 0.43  (0.23, 0.62) |
| Kingdom of Bhutan | 1453.53  (2098.94, 986.36) | 1026.57  (1471.42, 701.30) | 3795.14  (5491.94, 2574.60) | 1821.77  (2635.66, 1236.37) | 2.11  (1.99, 2.24) |
| Kingdom of Cambodia | 55630.38  (80635.89, 37813.13) | 2233.34  (3234.66, 1521.74) | 175037.67  (249683.50, 120462.34) | 3852.40  (5494.77, 2652.31) | 1.88  (1.85, 1.92) |
| Kingdom of Denmark | 63406.76  (89891.46, 43624.38) | 4836.97  (6856.16, 3325.44) | 78687.91  (110894.22, 54299.25) | 6227.72  (8781.31, 4296.80) | 0.79  (0.66, 0.92) |
| Kingdom of Eswatini | 3354.22  (4909.75, 2252.12) | 1729.44  (2522.90, 1166.53) | 6804.20  (9657.01, 4628.02) | 2145.53  (3042.39, 1459.37) | 0.42  (0.17, 0.68) |
| Kingdom of Lesotho | 4501.72  (6519.46, 3043.02) | 1197.50  (1730.19, 812.62) | 9092.54  (13239.35, 6157.43) | 1789.18  (2601.78, 1214.95) | 1.25  (1.12, 1.38) |
| Kingdom of Morocco | 183651.20  (261962.97, 124846.12) | 2914.65  (4168.94, 1982.65) | 375834.95  (545398.96, 257266.66) | 3880.30  (5631.03, 2655.36) | 1.01  (0.97, 1.05) |
| Kingdom of Norway | 56733.99  (80192.57, 39393.46) | 5353.83  (7570.27, 3713.30) | 74512.97  (105479.31, 51801.42) | 6138.26  (8699.41, 4261.95) | 0.32  (0.22, 0.43) |
| Kingdom of Saudi Arabia | 115260.99  (166550.34, 78299.97) | 3525.16  (5083.70, 2399.73) | 507688.81  (733933.77, 346367.30) | 4975.37  (7197.71, 3389.93) | 1.22  (1.19, 1.24) |
| Kingdom of Spain | 476732.48  (672791.77, 329511.57) | 4955.51  (6995.80, 3424.61) | 622267.00  (877880.68, 429187.29) | 6294.89  (8876.96, 4337.06) | 0.66  (0.53, 0.80) |
| Kingdom of Sweden | 84771.22  (121868.03, 58227.85) | 4116.45  (5916.26, 2821.95) | 109845.62  (156279.92, 75380.23) | 4980.61  (7086.13, 3411.68) | 0.49  (0.23, 0.74) |
| Kingdom of Thailand | 551220.89  (794720.89, 378531.87) | 3435.49  (4952.29, 2358.78) | 1082501.93  (1553820.09, 755843.68) | 6659.42  (9552.96, 4637.85) | 2.24  (2.08, 2.40) |
| Kingdom of the Netherlands | 189828.95  (269485.27, 129307.75) | 4754.12  (6750.62, 3235.47) | 220658.64  (314902.64, 151694.70) | 6001.76  (8576.83, 4119.29) | 0.70  (0.65, 0.74) |
| Kingdom of Tonga | 862.65  (1249.35, 587.02) | 3893.91  (5640.69, 2658.47) | 1338.35  (1930.91, 907.19) | 5318.19  (7677.86, 3604.59) | 0.69  (0.48, 0.91) |
| Kyrgyz Republic | 6803.19  (9907.33, 4535.40) | 647.45  (938.63, 433.87) | 13474.65  (19592.19, 8888.14) | 777.95  (1131.53, 512.85) | 0.45  (0.37, 0.53) |
| Lao People's Democratic Republic | 24111.33  (34378.06, 16386.21) | 2516.51  (3589.99, 1716.80) | 92987.99  (133310.83, 64551.68) | 4667.77  (6691.83, 3241.32) | 2.22  (2.16, 2.28) |
| Lebanese Republic | 25433.58  (36633.45, 17234.93) | 3388.97  (4883.07, 2297.48) | 66303.38  (95707.01, 45486.03) | 4456.87  (6435.80, 3054.64) | 0.89  (0.87, 0.91) |
| Malaysia | 206107.30  (298862.70, 142788.27) | 4597.16  (6668.26, 3189.14) | 668843.38  (950522.49, 461327.97) | 7877.54  (11194.77, 5429.38) | 1.88  (1.73, 2.04) |
| Mongolia | 3142.27  (4551.28, 2054.25) | 617.34  (890.59, 406.98) | 7455.12  (10713.24, 5012.51) | 879.31  (1267.28, 589.12) | 1.25  (1.20, 1.30) |
| Montenegro | 502.63  (764.20, 323.48) | 321.13  (488.28, 206.66) | 593.51  (878.37, 385.20) | 412.15  (613.38, 266.10) | 1.03  (0.94, 1.11) |
| New Zealand | 96735.13  (137018.91, 67478.65) | 10676.30  (15126.41, 7445.74) | 130184.61  (181781.28, 91856.74) | 10847.39  (15149.89, 7646.21) | -0.10  (-0.22, 0.01) |
| North Macedonia | 1369.67  (2141.74, 864.72) | 268.59  (420.14, 169.47) | 1996.11  (3002.91, 1297.02) | 373.65  (565.59, 241.17) | 1.16  (1.09, 1.24) |
| Northern Mariana Islands | 569.31  (809.68, 387.05) | 4056.88  (5768.47, 2758.49) | 589.07  (835.80, 400.57) | 5245.34  (7441.85, 3565.21) | 0.66  (0.50, 0.82) |
| Palestine | 12937.14  (18688.29, 8729.32) | 2928.06  (4220.76, 1983.90) | 48482.96  (69442.11, 32981.51) | 3713.23  (5322.37, 2529.61) | 0.75  (0.70, 0.80) |
| People's Democratic Republic of Algeria | 167734.98  (241565.11, 113923.49) | 2879.72  (4144.30, 1963.20) | 473937.79  (679629.95, 321831.60) | 4213.51  (6046.05, 2861.57) | 1.40  (1.36, 1.45) |
| People's Republic of Bangladesh | 173220.76  (253002.50, 115840.99) | 713.93  (1031.12, 481.02) | 543248.66  (774470.56, 369037.79) | 1178.89  (1679.62, 801.19) | 1.88  (1.74, 2.01) |
| People's Republic of China | 5127458.64  (7270274.81, 3550736.65) | 1600.46  (2264.96, 1110.71) | 9481520.05  (13488881.10, 6591669.74) | 2959.21  (4213.17, 2049.56) | 2.07  (1.91, 2.24) |
| Plurinational State of Bolivia | 64629.19  (92914.15, 43241.38) | 4239.97  (6091.54, 2839.36) | 178506.58  (253456.18, 119602.28) | 5703.66  (8100.56, 3820.50) | 1.00  (0.92, 1.08) |
| Portuguese Republic | 115804.52  (165279.21, 79630.50) | 4584.53  (6543.71, 3152.15) | 144967.89  (205814.43, 99307.45) | 6230.59  (8850.11, 4257.31) | 0.74  (0.55, 0.92) |
| Principality of Andorra | 833.48  (1185.14, 566.63) | 5512.42  (7840.44, 3750.72) | 1363.42  (1929.25, 939.30) | 6819.98  (9646.27, 4695.82) | 0.64  (0.54, 0.75) |
| Principality of Monaco | 413.91  (589.04, 284.12) | 5888.90  (8390.24, 4027.83) | 494.77  (698.16, 339.87) | 6935.51  (9790.87, 4755.86) | 0.48  (0.41, 0.55) |
| Puerto Rico | 32285.84  (46547.96, 21614.87) | 3371.60  (4861.60, 2257.85) | 32070.21  (46550.02, 21100.60) | 4303.67  (6248.61, 2827.34) | 0.82  (0.74, 0.90) |
| Republic of Albania | 2311.80  (3547.98, 1474.50) | 276.79  (423.22, 177.66) | 2241.89  (3358.24, 1465.78) | 364.43  (546.81, 237.51) | 0.86  (0.76, 0.95) |
| Republic of Angola | 17769.07  (25662.51, 11785.91) | 782.54  (1124.47, 522.52) | 107215.78  (156557.82, 71316.74) | 1407.11  (2045.79, 940.01) | 1.80  (1.65, 1.95) |
| Republic of Armenia | 5384.86  (7865.12, 3608.32) | 614.06  (895.99, 411.39) | 6786.63  (9911.48, 4563.94) | 912.99  (1337.51, 611.24) | 1.43  (1.38, 1.49) |
| Republic of Austria | 141884.48  (196154.44, 99995.85) | 7113.87  (9841.30, 5009.86) | 149421.53  (211072.88, 102831.11) | 7515.50  (10643.50, 5167.94) | -0.05  (-0.13, 0.03) |
| Republic of Azerbaijan | 11829.82  (17549.97, 7883.80) | 625.79  (924.83, 418.15) | 26693.60  (38871.44, 17757.93) | 965.52  (1409.11, 640.19) | 1.73  (1.61, 1.85) |
| Republic of Belarus | 9777.07  (14481.46, 6507.59) | 381.79  (566.92, 253.38) | 10945.32  (16171.29, 7368.69) | 508.97  (757.64, 338.42) | 1.12  (1.04, 1.20) |
| Republic of Benin | 9597.74  (13900.91, 6487.45) | 878.92  (1265.88, 597.89) | 52041.35  (74816.91, 34851.00) | 1608.92  (2309.23, 1080.11) | 1.85  (1.57, 2.13) |
| Republic of Botswana | 4193.73  (6066.72, 2843.32) | 1316.72  (1895.61, 896.43) | 14145.94  (20207.71, 9577.30) | 2066.34  (2952.68, 1398.36) | 1.67  (1.46, 1.89) |
| Republic of Bulgaria | 6741.86  (10292.17, 4295.94) | 325.35  (497.83, 206.44) | 5925.79  (8796.78, 3899.89) | 417.23  (623.57, 272.36) | 0.82  (0.78, 0.86) |
| Republic of Burundi | 10450.73  (14977.06, 7011.41) | 835.78  (1192.72, 564.42) | 27323.13  (39224.84, 18272.53) | 884.19  (1264.18, 593.73) | 0.09  (0.02, 0.17) |
| Republic of Cabo Verde | 752.18  (1087.22, 508.73) | 956.92  (1371.31, 653.96) | 2421.50  (3530.24, 1638.10) | 1602.44  (2335.39, 1084.46) | 1.45  (1.23, 1.67) |
| Republic of Cameroon | 31216.89  (45844.77, 20641.85) | 1316.26  (1926.61, 876.64) | 138280.49  (202780.97, 91131.98) | 1765.28  (2580.57, 1167.93) | 0.74  (0.64, 0.84) |
| Republic of Chad | 8543.02  (12514.39, 5738.75) | 652.51  (949.11, 442.16) | 37890.53  (54841.87, 25442.31) | 992.59  (1429.00, 670.62) | 0.90  (0.65, 1.14) |
| Republic of Chile | 85927.80  (125212.18, 59210.09) | 2372.70  (3456.36, 1634.91) | 194117.18  (280986.75, 133452.62) | 4085.53  (5913.41, 2807.86) | 1.71  (1.40, 2.03) |
| Republic of Colombia | 275130.56  (396292.31, 183689.24) | 3154.44  (4542.70, 2101.86) | 544009.91  (789558.70, 368772.98) | 4133.29  (6002.71, 2800.41) | 0.79  (0.73, 0.85) |
| Republic of Costa Rica | 30739.17  (44823.45, 20612.08) | 3912.89  (5704.36, 2628.66) | 67301.93  (97117.69, 44977.06) | 5173.80  (7466.21, 3455.52) | 0.80  (0.74, 0.86) |
| Republic of Croatia | 3604.38  (5487.27, 2331.42) | 298.49  (456.38, 192.23) | 3629.79  (5455.56, 2376.24) | 403.90  (610.13, 262.67) | 1.07  (0.97, 1.17) |
| Republic of Cuba | 75590.91  (108382.57, 50518.89) | 2460.42  (3525.90, 1646.79) | 80618.41  (115506.24, 53742.70) | 3268.01  (4681.63, 2175.31) | 0.94  (0.89, 0.99) |
| Republic of Cyprus | 8687.89  (12258.83, 5992.00) | 4377.30  (6178.03, 3017.82) | 22901.00  (32832.33, 15696.44) | 6321.90  (9087.59, 4326.37) | 1.31  (1.14, 1.48) |
| Republic of C么te d'Ivoire | 24341.33  (35510.57, 16231.91) | 895.07  (1297.89, 600.21) | 98278.49  (144273.86, 66267.13) | 1474.26  (2158.14, 996.69) | 1.41  (1.13, 1.70) |
| Republic of Djibouti | 956.38  (1401.17, 645.47) | 985.57  (1432.57, 670.58) | 5406.93  (7926.00, 3625.93) | 1675.39  (2456.37, 1122.87) | 1.81  (1.67, 1.96) |
| Republic of Ecuador | 135157.69  (189808.25, 92786.50) | 5349.53  (7510.99, 3666.98) | 333593.17  (463733.11, 232247.70) | 7052.64  (9805.99, 4906.80) | 0.88  (0.64, 1.11) |
| Republic of El Salvador | 41815.99  (61340.82, 28112.76) | 3195.13  (4683.25, 2148.68) | 80762.69  (117157.39, 54852.60) | 4530.90  (6574.04, 3076.02) | 1.06  (0.95, 1.16) |
| Republic of Equatorial Guinea | 871.55  (1284.90, 588.94) | 893.98  (1310.40, 608.05) | 7616.72  (10935.81, 5108.75) | 2089.87  (2997.69, 1404.15) | 2.98  (2.53, 3.43) |
| Republic of Estonia | 1565.19  (2312.03, 1045.84) | 407.28  (604.14, 270.85) | 1619.54  (2405.16, 1077.28) | 577.43  (863.94, 380.94) | 1.42  (1.33, 1.51) |
| Republic of Fiji | 6013.18  (8777.23, 4090.26) | 3068.75  (4482.74, 2089.98) | 10714.00  (15342.24, 7349.87) | 4688.94  (6714.67, 3216.32) | 1.24  (1.09, 1.38) |
| Republic of Finland | 63928.17  (90895.36, 43745.52) | 5002.47  (7114.86, 3416.88) | 72406.47  (102256.80, 49742.03) | 6342.16  (8968.38, 4357.37) | 0.70  (0.65, 0.74) |
| Republic of Ghana | 32704.77  (47287.85, 21954.43) | 932.02  (1342.24, 628.48) | 127752.36  (185100.60, 87133.47) | 1395.60  (2018.73, 952.90) | 0.93  (0.68, 1.18) |
| Republic of Guatemala | 49938.57  (72626.89, 33697.79) | 2725.46  (3967.62, 1839.83) | 172329.99  (252729.12, 113648.57) | 3904.87  (5719.42, 2576.25) | 0.96  (0.84, 1.07) |
| Republic of Guinea | 11073.66  (15962.90, 7438.16) | 814.92  (1170.20, 550.11) | 40959.72  (59398.25, 27992.03) | 1235.53  (1787.25, 847.10) | 1.11  (0.97, 1.25) |
| Republic of Guinea-Bissau | 1881.95  (2792.04, 1263.36) | 815.49  (1202.75, 551.32) | 6477.83  (9385.69, 4365.03) | 1233.64  (1780.11, 834.36) | 1.05  (0.78, 1.32) |
| Republic of Guyana | 4089.26  (6014.23, 2722.75) | 1993.36  (2922.35, 1331.52) | 5881.48  (8456.25, 3934.88) | 2886.18  (4151.73, 1931.13) | 1.17  (1.07, 1.27) |
| Republic of Haiti | 23189.85  (34040.62, 15279.94) | 1504.48  (2204.80, 993.83) | 66177.94  (94409.43, 44808.31) | 1862.16  (2655.67, 1261.06) | 0.83  (0.78, 0.88) |
| Republic of Honduras | 27669.99  (40615.68, 18613.11) | 2622.23  (3841.75, 1768.04) | 112487.90  (164230.95, 76151.37) | 3942.76  (5757.31, 2672.75) | 1.27  (1.16, 1.38) |
| Republic of Iceland | 3704.11  (5282.53, 2560.67) | 5680.88  (8102.77, 3927.29) | 5689.70  (8096.80, 3950.05) | 7109.79  (10120.19, 4926.57) | 0.74  (0.69, 0.79) |
| Republic of India | 2610616.32  (3621347.83, 1854544.21) | 1297.64  (1796.51, 924.43) | 9198653.67  (12904729.33, 6486342.48) | 2429.87  (3409.12, 1713.86) | 2.35  (2.20, 2.50) |
| Republic of Indonesia | 1377477.18  (1962118.89, 957370.21) | 2885.24  (4104.76, 2009.51) | 4160507.64  (5909126.25, 2913699.92) | 5510.68  (7827.70, 3855.35) | 2.37  (2.28, 2.46) |
| Republic of Iraq | 137083.38  (200005.15, 94067.74) | 3375.77  (4913.16, 2321.32) | 409469.50  (592943.20, 278687.79) | 3881.34  (5618.46, 2644.00) | 0.61  (0.49, 0.73) |
| Republic of Italy | 2300243.75  (3199200.84, 1621926.12) | 16092.94  (22389.82, 11336.93) | 1838181.55  (2561275.00, 1285146.20) | 15307.74  (21343.59, 10698.08) | -0.42  (-0.54, -0.29) |
| Republic of Kazakhstan | 29144.48  (42887.29, 19201.45) | 703.53  (1035.00, 463.42) | 47128.96  (67337.30, 31720.69) | 992.21  (1419.88, 666.36) | 1.21  (1.18, 1.25) |
| Republic of Kenya | 58309.56  (83872.51, 39610.97) | 1151.23  (1643.03, 791.39) | 188159.98  (270945.81, 128629.48) | 1430.64  (2048.44, 983.28) | 0.53  (0.42, 0.64) |
| Republic of Kiribati | 492.01  (704.33, 336.48) | 2616.77  (3752.14, 1790.49) | 1236.32  (1742.62, 852.26) | 3855.72  (5435.91, 2659.56) | 1.12  (0.90, 1.34) |
| Republic of Korea | 492741.91  (707182.36, 333447.36) | 3898.01  (5592.97, 2639.47) | 711995.18  (1023485.24, 486960.23) | 6038.89  (8659.97, 4131.52) | 1.11  (0.77, 1.45) |
| Republic of Latvia | 2635.19  (3970.40, 1756.98) | 404.08  (611.58, 268.11) | 2085.21  (3000.98, 1415.83) | 530.92  (771.01, 357.33) | 1.08  (1.00, 1.15) |
| Republic of Liberia | 5153.77  (7557.24, 3453.96) | 932.34  (1357.87, 627.91) | 18944.18  (27560.56, 12777.87) | 1363.87  (1976.70, 923.09) | 1.40  (1.27, 1.52) |
| Republic of Lithuania | 3408.11  (5074.69, 2219.73) | 367.88  (549.12, 238.91) | 2955.53  (4419.92, 1994.81) | 511.61  (769.97, 342.06) | 1.30  (1.21, 1.38) |
| Republic of Madagascar | 24566.86  (35644.37, 16528.39) | 921.30  (1326.76, 624.41) | 76585.28  (111005.13, 51473.76) | 1064.57  (1536.13, 719.94) | 0.53  (0.48, 0.58) |
| Republic of Malawi | 27858.63  (40606.86, 18538.18) | 1238.62  (1791.75, 829.60) | 68529.15  (99315.51, 46499.83) | 1377.41  (1985.09, 938.96) | 0.44  (0.32, 0.57) |
| Republic of Maldives | 1283.57  (1839.25, 873.88) | 2723.59  (3906.76, 1860.68) | 7368.23  (10567.26, 5074.40) | 6307.02  (9061.94, 4333.53) | 3.39  (3.10, 3.67) |
| Republic of Mali | 13241.33  (19179.58, 8997.32) | 696.37  (1002.31, 476.38) | 60112.11  (87048.99, 40293.43) | 1110.53  (1598.46, 749.76) | 1.25  (1.04, 1.47) |
| Republic of Malta | 4816.59  (6874.46, 3328.60) | 5062.75  (7223.22, 3497.15) | 6525.60  (9298.57, 4473.87) | 6858.63  (9790.52, 4688.34) | 0.91  (0.77, 1.05) |
| Republic of Mauritius | 13545.50  (19920.47, 9377.18) | 4492.62  (6609.19, 3110.15) | 22837.49  (32466.63, 15872.61) | 7204.68  (10251.44, 5002.82) | 1.67  (1.54, 1.79) |
| Republic of Moldova | 3653.57  (5471.96, 2379.25) | 321.51  (482.83, 209.06) | 4245.38  (6185.06, 2866.27) | 472.95  (696.08, 315.19) | 1.52  (1.37, 1.67) |
| Republic of Mozambique | 28138.65  (41097.64, 18998.99) | 901.62  (1309.72, 612.74) | 102859.45  (148663.75, 68878.33) | 1364.10  (1959.68, 918.53) | 1.33  (1.25, 1.42) |
| Republic of Namibia | 4241.44  (6045.66, 2897.49) | 1263.46  (1797.73, 868.28) | 11223.84  (16231.01, 7703.88) | 1693.89  (2446.55, 1164.20) | 0.92  (0.79, 1.05) |
| Republic of Nauru | 81.23  (117.15, 55.13) | 3332.10  (4811.60, 2266.39) | 137.50  (200.02, 94.14) | 4827.54  (7022.42, 3310.93) | 1.06  (0.99, 1.12) |
| Republic of Nicaragua | 26900.02  (39530.23, 17944.31) | 2976.97  (4366.59, 1992.42) | 77229.18  (110732.30, 51379.24) | 4240.35  (6077.79, 2822.03) | 1.05  (0.96, 1.14) |
| Republic of Niue | 17.88  (25.48, 12.08) | 3632.86  (5173.95, 2456.35) | 21.02  (30.33, 14.37) | 5514.80  (7955.70, 3767.48) | 1.26  (1.10, 1.43) |
| Republic of Palau | 156.58  (226.00, 106.40) | 3810.34  (5498.67, 2592.89) | 202.60  (291.58, 138.35) | 5501.51  (7922.00, 3751.82) | 1.04  (0.87, 1.20) |
| Republic of Panama | 16939.08  (24812.03, 11322.32) | 2754.50  (4030.88, 1843.33) | 47083.79  (69087.68, 31691.67) | 4403.74  (6461.78, 2963.54) | 1.36  (1.27, 1.45) |
| Republic of Paraguay | 6816.38  (10154.25, 4459.07) | 730.41  (1084.49, 481.90) | 21774.98  (32083.02, 14414.11) | 1145.87  (1687.55, 758.91) | 1.67  (1.61, 1.73) |
| Republic of Peru | 233536.06  (331316.97, 155560.94) | 4304.77  (6104.04, 2869.57) | 593075.07  (867961.93, 401485.73) | 6134.63  (8983.03, 4150.05) | 1.24  (1.20, 1.28) |
| Republic of Poland | 46198.85  (68870.56, 30149.07) | 487.05  (728.65, 316.48) | 45296.45  (61737.97, 31826.88) | 513.10  (702.95, 358.28) | -0.01  (-0.08, 0.06) |
| Republic of Rwanda | 17519.21  (25522.46, 11953.54) | 1088.36  (1581.24, 745.93) | 48432.41  (70825.22, 32725.02) | 1377.45  (2008.44, 933.23) | 0.81  (0.75, 0.88) |
| Republic of San Marino | 351.95  (502.05, 241.20) | 5632.10  (8037.06, 3857.78) | 475.01  (668.34, 324.06) | 6704.95  (9440.71, 4569.41) | 0.52  (0.43, 0.60) |
| Republic of Senegal | 17905.04  (26187.51, 12143.36) | 1050.25  (1526.29, 717.48) | 51595.64  (74497.88, 34848.94) | 1325.73  (1907.21, 897.11) | 0.52  (0.39, 0.64) |
| Republic of Serbia | 6509.98  (9905.23, 4122.78) | 278.37  (424.29, 175.89) | 7688.83  (11222.42, 4999.25) | 377.84  (554.14, 244.71) | 1.11  (1.05, 1.18) |
| Republic of Seychelles | 866.82  (1254.34, 605.25) | 4793.17  (6930.87, 3350.19) | 1629.99  (2315.23, 1128.50) | 6738.58  (9572.58, 4663.83) | 1.08  (1.02, 1.14) |
| Republic of Sierra Leone | 8060.51  (11508.80, 5400.38) | 804.79  (1144.07, 542.80) | 31031.72  (45580.16, 20849.01) | 1366.94  (1999.28, 922.07) | 1.53  (1.39, 1.67) |
| Republic of Singapore | 42252.29  (60740.71, 28962.76) | 4448.39  (6393.35, 3052.25) | 107710.00  (155548.30, 74321.35) | 7156.57  (10277.61, 4937.48) | 1.54  (1.39, 1.69) |
| Republic of Slovenia | 1522.55  (2314.02, 991.20) | 303.72  (462.48, 196.96) | 1795.86  (2654.80, 1168.73) | 426.34  (633.90, 275.90) | 1.16  (1.05, 1.27) |
| Republic of South Africa | 169239.08  (245156.01, 114993.95) | 1759.86  (2541.84, 1200.49) | 351648.30  (504933.27, 240701.27) | 2254.18  (3239.34, 1541.10) | 0.93  (0.85, 1.00) |
| Republic of South Sudan | 12987.97  (18948.16, 8745.01) | 1011.32  (1462.69, 686.43) | 25758.49  (37578.41, 17353.79) | 1124.47  (1631.09, 761.63) | 0.18  (0.07, 0.29) |
| Republic of Sudan | 91334.99  (131487.10, 62033.42) | 1958.08  (2812.45, 1331.71) | 393148.98  (562865.31, 271121.63) | 3477.76  (4977.00, 2400.42) | 1.95  (1.80, 2.11) |
| Republic of Suriname | 2164.75  (3086.00, 1461.68) | 2222.39  (3161.85, 1504.26) | 4392.46  (6324.01, 2934.92) | 3032.21  (4365.82, 2025.34) | 0.99  (0.95, 1.03) |
| Republic of Tajikistan | 6196.55  (9169.11, 4118.52) | 516.73  (758.78, 346.35) | 17040.85  (24689.15, 11204.89) | 665.58  (963.57, 437.87) | 0.90  (0.83, 0.96) |
| Republic of the Congo | 6074.02  (8845.72, 4079.75) | 1091.38  (1582.58, 739.88) | 21728.82  (31480.20, 14675.60) | 1520.75  (2200.93, 1029.12) | 0.90  (0.76, 1.04) |
| Republic of the Gambia | 2032.29  (2930.27, 1364.56) | 900.98  (1291.74, 609.74) | 8249.58  (12033.31, 5627.68) | 1344.74  (1948.59, 921.72) | 1.03  (0.86, 1.19) |
| Republic of the Marshall Islands | 217.83  (312.52, 147.47) | 2234.75  (3200.79, 1518.22) | 558.26  (795.16, 379.31) | 3772.05  (5372.34, 2564.59) | 1.47  (1.29, 1.65) |
| Republic of the Niger | 12549.77  (17831.39, 8441.41) | 733.07  (1034.70, 497.61) | 51985.56  (75508.82, 34978.99) | 992.13  (1431.49, 671.34) | 0.91  (0.76, 1.05) |
| Republic of the Philippines | 486354.91  (695144.59, 336910.31) | 3159.86  (4512.10, 2193.45) | 1573840.57  (2244798.19, 1094236.39) | 5366.33  (7654.25, 3733.00) | 2.17  (2.02, 2.31) |
| Republic of the Union of Myanmar | 241141.61  (348557.76, 165112.25) | 2321.66  (3352.40, 1589.88) | 719359.02  (1013153.98, 499225.63) | 4766.35  (6713.01, 3308.13) | 2.76  (2.62, 2.90) |
| Republic of Trinidad and Tobago | 6916.26  (9889.69, 4602.51) | 2238.19  (3198.11, 1490.19) | 11012.59  (15823.08, 7355.06) | 3245.88  (4673.03, 2164.83) | 1.32  (1.17, 1.46) |
| Republic of Tunisia | 57936.48  (84857.50, 38968.45) | 2824.65  (4131.82, 1904.17) | 121990.37  (173965.66, 84013.32) | 3978.43  (5675.02, 2736.75) | 1.21  (1.17, 1.26) |
| Republic of Turkey | 380697.07  (552912.67, 258200.64) | 2665.92  (3870.87, 1812.29) | 807611.92  (1149177.47, 553821.11) | 3735.04  (5317.71, 2559.60) | 1.18  (1.12, 1.24) |
| Republic of Uganda | 39430.66  (57180.93, 26561.61) | 1035.67  (1486.60, 702.58) | 133359.62  (192460.85, 90951.14) | 1287.88  (1849.69, 881.20) | 0.72  (0.69, 0.76) |
| Republic of Uzbekistan | 32884.47  (48201.46, 21698.14) | 671.08  (979.53, 445.14) | 84081.06  (118739.75, 55865.73) | 938.68  (1328.35, 622.54) | 1.15  (1.02, 1.28) |
| Republic of Vanuatu | 900.07  (1292.18, 615.72) | 2566.13  (3681.30, 1760.20) | 3008.10  (4366.23, 2043.63) | 3834.48  (5565.57, 2608.78) | 1.21  (1.16, 1.27) |
| Republic of Yemen | 55289.41  (80630.27, 37871.33) | 2021.24  (2937.81, 1389.58) | 208737.99  (302144.71, 140738.17) | 2500.45  (3610.80, 1690.99) | 0.99  (0.86, 1.12) |
| Republic of Zambia | 25290.78  (37256.27, 16773.54) | 1388.42  (2029.68, 928.23) | 83189.60  (121351.41, 55453.68) | 1691.37  (2455.00, 1132.23) | 0.51  (0.46, 0.56) |
| Republic of Zimbabwe | 34718.86  (50205.86, 23197.98) | 1458.08  (2101.69, 978.02) | 63881.10  (91343.77, 43259.12) | 1575.34  (2247.71, 1069.09) | -0.12  (-0.31, 0.06) |
| Romania | 15629.81  (23831.57, 9872.67) | 278.08  (424.01, 175.70) | 16024.61  (24110.09, 10560.23) | 396.30  (600.38, 259.51) | 1.24  (1.19, 1.29) |
| Russian Federation | 152313.72  (221425.32, 102158.88) | 403.46  (590.01, 269.37) | 181821.94  (263376.97, 125430.72) | 523.36  (766.63, 356.35) | 1.00  (0.96, 1.04) |
| Saint Kitts and Nevis | 257.87  (365.79, 173.80) | 2559.34  (3622.33, 1730.16) | 541.71  (789.68, 364.48) | 3476.08  (5070.45, 2335.04) | 0.90  (0.81, 0.98) |
| Saint Lucia | 726.94  (1046.36, 486.38) | 2125.05  (3055.05, 1428.54) | 1299.89  (1825.27, 871.31) | 2854.98  (4013.40, 1910.16) | 0.68  (0.54, 0.82) |
| Saint Vincent and the Grenadines | 512.69  (733.80, 340.74) | 1941.01  (2762.28, 1297.39) | 810.97  (1169.95, 542.00) | 2926.26  (4221.93, 1954.56) | 1.37  (1.27, 1.47) |
| Slovak Republic | 3823.09  (5763.09, 2451.64) | 287.22  (433.55, 183.81) | 5068.03  (7572.80, 3351.81) | 399.95  (601.29, 262.18) | 1.07  (1.03, 1.12) |
| Socialist Republic of Viet Nam | 371449.48  (528413.45, 255600.57) | 2181.30  (3097.22, 1500.65) | 1124756.10  (1602273.97, 776975.62) | 4371.36  (6229.93, 3018.74) | 2.80  (2.61, 2.98) |
| Solomon Islands | 1637.89  (2367.61, 1114.69) | 2220.19  (3208.97, 1515.82) | 5974.04  (8490.94, 4048.02) | 3492.48  (4963.72, 2370.50) | 1.23  (0.98, 1.47) |
| State of Eritrea | 5741.59  (8289.03, 3884.44) | 738.67  (1056.84, 502.92) | 18274.40  (26393.90, 12241.71) | 1107.49  (1594.10, 743.84) | 1.27  (1.06, 1.47) |
| State of Israel | 56541.15  (81544.59, 39165.34) | 4642.38  (6684.70, 3219.76) | 133420.24  (189450.00, 92063.31) | 6012.38  (8535.26, 4148.61) | 0.73  (0.65, 0.82) |
| State of Kuwait | 18016.72  (26052.46, 12167.30) | 4289.61  (6207.30, 2901.02) | 75833.60  (108288.23, 51769.56) | 5169.37  (7376.48, 3523.96) | 0.80  (0.74, 0.86) |
| State of Libya | 34837.61  (50268.68, 23429.47) | 3826.88  (5517.45, 2585.84) | 84103.46  (120158.13, 57811.40) | 4233.95  (6049.89, 2908.67) | 0.46  (0.42, 0.51) |
| State of Qatar | 3427.37  (5026.22, 2345.64) | 4290.64  (6292.43, 2936.01) | 28425.48  (41110.42, 19347.61) | 5075.53  (7343.35, 3448.38) | 0.54  (0.50, 0.58) |
| Sultanate of Oman | 9450.40  (13785.63, 6339.51) | 2781.87  (4052.40, 1873.63) | 47063.56  (67452.82, 31972.76) | 4600.78  (6595.14, 3124.43) | 1.74  (1.65, 1.82) |
| Swiss Confederation | 93997.31  (134910.55, 64470.03) | 5289.81  (7596.01, 3624.83) | 117519.84  (167163.64, 81166.37) | 5959.38  (8488.14, 4110.32) | 0.41  (0.40, 0.43) |
| Syrian Arab Republic | 80314.41  (113730.02, 54459.60) | 2916.71  (4128.29, 1981.59) | 148807.89  (214946.41, 101138.68) | 3873.00  (5594.09, 2641.05) | 0.99  (0.91, 1.07) |
| Taiwan (Province of China) | 163582.39  (235058.61, 109527.28) | 2944.61  (4237.01, 1973.39) | 268089.61  (368287.01, 189441.13) | 4753.67  (6536.85, 3344.72) | 1.78  (1.70, 1.86) |
| Togolese Republic | 7097.00  (10356.91, 4770.94) | 838.45  (1216.67, 567.24) | 27663.30  (40278.54, 18687.50) | 1283.46  (1865.83, 868.74) | 1.09  (0.92, 1.26) |
| Tokelau | 10.70  (15.31, 7.31) | 3044.35  (4353.67, 2083.25) | 15.96  (22.85, 10.88) | 4988.32  (7139.72, 3403.03) | 1.49  (1.34, 1.64) |
| Turkmenistan | 5795.48  (8518.25, 3821.20) | 654.65  (958.08, 434.18) | 11993.36  (17522.82, 8033.77) | 953.55  (1392.89, 638.74) | 1.26  (1.23, 1.28) |
| Tuvalu | 69.44  (99.06, 47.60) | 2841.96  (4054.28, 1948.14) | 130.64  (187.38, 89.40) | 4502.03  (6459.78, 3084.80) | 1.26  (1.08, 1.44) |
| Ukraine | 48636.53  (71840.86, 32543.21) | 379.14  (562.68, 252.52) | 47337.53  (69147.33, 31962.17) | 454.13  (671.71, 301.85) | 0.79  (0.72, 0.87) |
| Union of the Comoros | 1298.13  (1940.28, 867.03) | 1243.99  (1842.45, 837.84) | 2882.12  (4159.96, 1945.18) | 1477.86  (2130.61, 999.86) | 0.31  (0.14, 0.47) |
| United Arab Emirates | 12252.40  (17702.48, 8368.69) | 3527.31  (5094.01, 2408.97) | 79711.31  (116344.63, 54793.99) | 4644.27  (6775.54, 3183.76) | 0.85  (0.73, 0.96) |
| United Kingdom of Great Britain and Northern Ireland | 835302.67  (1186848.13, 577920.91) | 5845.85  (8311.36, 4039.86) | 1125560.66  (1596041.19, 783161.35) | 7306.96  (10369.41, 5077.77) | 0.48  (0.35, 0.61) |
| United Mexican States | 1473996.20  (2072475.81, 1011016.46) | 6742.59  (9473.96, 4636.41) | 2379831.54  (3309872.08, 1659967.28) | 6800.66  (9458.27, 4741.46) | -0.53  (-0.77, -0.28) |
| United Republic of Tanzania | 71601.20  (104684.73, 47730.17) | 1205.24  (1751.34, 810.15) | 217233.48  (313310.70, 145912.83) | 1467.31  (2110.59, 988.75) | 0.63  (0.54, 0.72) |
| United States of America | 4080725.01  (5783209.23, 2860223.87) | 6017.23  (8536.01, 4212.07) | 5774954.04  (7698228.66, 4267680.35) | 7599.79  (10130.88, 5617.03) | -0.58  (-1.12, -0.04) |
| United States Virgin Islands | 933.33  (1372.58, 624.98) | 3268.25  (4806.07, 2188.10) | 702.66  (1013.71, 466.92) | 4128.47  (5958.45, 2737.71) | 0.71  (0.61, 0.82) |

Abbreviations: ASPR, age standardized prevalence rate; EAPC, estimated annual percentage change; SDI, socio-demographic index; UI, uncertainty interval; CI, confidence interval.
