# Supplementary material for: Global burden of polycystic ovary syndrome in women of reproductive age, 1990–2021: Analysis of the global burden of disease study 2021 with projections to 2050
Source: PLoS One. 2025 Oct 7;20(10):e0333000. doi: 10.1371/journal.pone.0333000 (PMC12503323; doi:10.1371/journal.pone.0333000)
Supplement: S5 Table — (DOCX) [file pone.0333000.s015.docx]

Table 6. DALYs of PCOS among women of reproductive age between 1990 and 2021 at the national level.

| Nations | 1990 | | 2021 | | 1990-2021 EAPC |
| --- | --- | --- | --- | --- | --- |
|  | All-age DALYs | ASDR per 100,000 population | All-age DALYs | ASDR per 100,000 population |  |
|  | n*10^3^ (95% UI) | n (95% UI) | n*10^3^ (95% UI) | n (95% UI) | n (95% CI) |
| American Samoa | 4.12  (8.60, 1.74) | 33.82  (70.70, 14.27) | 5.38  (11.32, 2.33) | 46.56  (98.06, 20.13) | 0.89  (0.71, 1.07) |
| Antigua and Barbuda | 3.28  (6.91, 1.37) | 19.96  (41.98, 8.35) | 6.36  (13.35, 2.73) | 26.50  (55.72, 11.39) | 0.80  (0.75, 0.86) |
| Arab Republic of Egypt | 4214.89  (8834.21, 1805.79) | 31.90  (66.90, 13.69) | 10291.56  (21542.56, 4517.99) | 39.46  (82.55, 17.33) | 0.52  (0.45, 0.60) |
| Argentine Republic | 1587.71  (3321.22, 687.45) | 19.86  (41.53, 8.60) | 3647.38  (7588.88, 1585.99) | 30.61  (63.72, 13.31) | 1.35  (1.17, 1.52) |
| Australia | 2866.82  (5826.98, 1261.69) | 63.98  (129.95, 28.15) | 4658.46  (9744.80, 2063.75) | 77.03  (160.76, 34.06) | 0.38  (0.26, 0.50) |
| Barbados | 17.25  (36.29, 7.30) | 24.97  (52.57, 10.57) | 20.63  (43.95, 8.78) | 29.32  (62.49, 12.50) | 0.47  (0.43, 0.50) |
| Belize | 8.57  (18.06, 3.66) | 20.09  (42.35, 8.57) | 34.90  (73.86, 14.82) | 28.77  (60.80, 12.22) | 0.94  (0.68, 1.20) |
| Bermuda | 5.20  (10.93, 2.17) | 30.29  (63.82, 12.64) | 4.45  (9.29, 1.88) | 33.65  (70.36, 14.29) | 0.24  (0.19, 0.30) |
| Bolivarian Republic of Venezuela | 1625.43  (3455.59, 691.74) | 33.21  (70.56, 14.12) | 2831.01  (5997.11, 1212.19) | 41.67  (88.19, 17.87) | 0.66  (0.59, 0.73) |
| Bosnia and Herzegovina | 24.01  (54.85, 8.88) | 2.05  (4.70, 0.76) | 23.19  (50.98, 9.10) | 3.18  (7.03, 1.24) | 1.57  (1.35, 1.79) |
| Brunei Darussalam | 28.14  (58.18, 12.37) | 41.20  (85.21, 18.12) | 81.26  (167.68, 36.36) | 64.19  (132.53, 28.69) | 1.50  (1.34, 1.65) |
| Burkina Faso | 153.20  (329.54, 63.77) | 7.30  (15.62, 3.04) | 632.21  (1347.28, 263.70) | 11.45  (24.31, 4.80) | 1.22  (1.03, 1.42) |
| Canada | 1853.82  (3820.25, 799.96) | 24.86  (51.30, 10.73) | 2643.50  (5519.03, 1135.38) | 31.79  (66.44, 13.65) | 0.72  (0.61, 0.82) |
| Central African Republic | 57.06  (123.30, 24.10) | 8.88  (19.11, 3.77) | 133.25  (283.81, 55.59) | 9.64  (20.51, 4.05) | 0.07  (-0.06, 0.19) |
| Commonwealth of Dominica | 3.41  (7.25, 1.44) | 19.85  (42.09, 8.40) | 4.46  (9.62, 1.91) | 27.17  (58.62, 11.62) | 0.85  (0.73, 0.97) |
| Commonwealth of the Bahamas | 18.60  (39.69, 7.76) | 25.32  (53.95, 10.61) | 32.32  (69.83, 13.95) | 30.16  (65.18, 13.02) | 0.51  (0.44, 0.59) |
| Cook Islands | 1.60  (3.40, 0.69) | 34.81  (73.86, 14.97) | 2.20  (4.68, 0.95) | 51.50  (109.37, 22.31) | 1.12  (0.99, 1.26) |
| Czech Republic | 68.59  (151.44, 26.20) | 2.68  (5.91, 1.02) | 77.92  (165.17, 30.83) | 3.44  (7.25, 1.35) | 0.76  (0.70, 0.82) |
| Democratic People's Republic of Korea | 824.36  (1759.50, 346.31) | 14.52  (30.96, 6.09) | 1072.03  (2247.65, 453.10) | 16.28  (34.17, 6.88) | 0.33  (0.25, 0.42) |
| Democratic Republic of Sao Tome and Principe | 2.14  (4.63, 0.88) | 8.39  (18.07, 3.46) | 7.01  (14.83, 2.96) | 12.49  (26.39, 5.28) | 1.05  (0.84, 1.27) |
| Democratic Republic of the Congo | 610.44  (1282.14, 246.90) | 7.18  (15.01, 2.92) | 2243.16  (4716.68, 950.94) | 10.53  (22.12, 4.46) | 1.18  (0.99, 1.36) |
| Democratic Republic of Timor-Leste | 39.74  (81.34, 17.39) | 21.10  (43.25, 9.24) | 120.93  (250.05, 51.53) | 34.96  (72.13, 14.89) | 1.97  (1.86, 2.08) |
| Democratic Socialist Republic of Sri Lanka | 1504.26  (3118.03, 654.07) | 32.59  (67.64, 14.16) | 2993.02  (6186.46, 1302.46) | 53.25  (110.06, 23.15) | 1.83  (1.62, 2.05) |
| Dominican Republic | 333.29  (697.19, 142.35) | 17.51  (36.60, 7.49) | 791.71  (1694.97, 332.39) | 27.21  (58.28, 11.43) | 1.53  (1.44, 1.63) |
| Eastern Republic of Uruguay | 152.54  (319.91, 65.94) | 20.35  (42.68, 8.80) | 278.35  (574.86, 118.15) | 33.46  (69.13, 14.21) | 1.60  (1.40, 1.79) |
| Federal Democratic Republic of Ethiopia | 726.91  (1528.04, 305.60) | 6.52  (13.63, 2.75) | 2745.12  (5823.72, 1162.41) | 9.95  (21.14, 4.23) | 1.55  (1.49, 1.61) |
| Federal Democratic Republic of Nepal | 297.06  (626.31, 123.61) | 6.53  (13.78, 2.73) | 1030.63  (2159.10, 430.55) | 11.26  (23.55, 4.71) | 1.80  (1.76, 1.84) |
| Federal Republic of Germany | 8372.09  (17514.02, 3795.37) | 42.60  (89.28, 19.27) | 8990.12  (18538.59, 4000.30) | 52.57  (108.20, 23.35) | 0.60  (0.55, 0.65) |
| Federal Republic of Nigeria | 1787.44  (3747.65, 760.30) | 8.92  (18.67, 3.80) | 6997.31  (14997.62, 2958.79) | 12.28  (26.28, 5.20) | 0.76  (0.58, 0.94) |
| Federal Republic of Somalia | 132.96  (278.50, 55.31) | 7.88  (16.51, 3.28) | 446.20  (938.90, 182.47) | 9.25  (19.50, 3.79) | 0.62  (0.57, 0.67) |
| Federated States of Micronesia | 5.85  (12.46, 2.53) | 25.19  (53.49, 10.93) | 9.50  (19.50, 4.08) | 36.39  (74.72, 15.65) | 0.96  (0.75, 1.17) |
| Federative Republic of Brazil | 3691.18  (7928.06, 1542.18) | 9.44  (20.23, 3.95) | 5922.52  (12681.16, 2522.61) | 10.08  (21.62, 4.29) | -0.23  (-0.41, -0.05) |
| French Republic | 6427.98  (13583.28, 2805.87) | 44.25  (93.61, 19.33) | 7779.85  (16140.62, 3426.04) | 55.25  (114.71, 24.31) | 0.68  (0.64, 0.72) |
| Gabonese Republic | 23.55  (49.80, 9.88) | 10.63  (22.43, 4.47) | 78.66  (167.36, 33.15) | 16.00  (34.06, 6.75) | 1.18  (1.04, 1.33) |
| Georgia | 95.11  (203.60, 38.17) | 6.89  (14.77, 2.77) | 94.34  (197.02, 39.50) | 12.10  (25.27, 5.07) | 2.30  (2.05, 2.55) |
| Grand Duchy of Luxembourg | 47.74  (100.27, 20.87) | 48.48  (102.05, 21.17) | 96.51  (203.40, 41.62) | 61.83  (130.18, 26.62) | 0.79  (0.73, 0.84) |
| Greenland | 3.23  (6.68, 1.40) | 21.17  (43.72, 9.14) | 3.62  (7.56, 1.57) | 28.07  (58.67, 12.19) | 0.93  (0.85, 1.02) |
| Grenada | 3.36  (7.07, 1.41) | 17.10  (35.88, 7.17) | 6.27  (13.63, 2.72) | 24.49  (53.26, 10.60) | 1.00  (0.87, 1.13) |
| Guam | 13.20  (27.78, 5.58) | 37.33  (78.60, 15.78) | 18.79  (39.40, 8.14) | 52.35  (109.82, 22.68) | 1.11  (1.01, 1.20) |
| Hashemite Kingdom of Jordan | 238.96  (499.63, 100.82) | 28.24  (58.95, 11.95) | 1092.29  (2325.65, 466.80) | 35.12  (74.71, 15.01) | 0.79  (0.70, 0.88) |
| Hellenic Republic | 1246.99  (2612.84, 545.19) | 49.48  (103.64, 21.63) | 1320.58  (2784.49, 581.20) | 61.67  (130.22, 27.13) | 0.51  (0.33, 0.69) |
| Hungary | 72.97  (163.22, 28.44) | 2.89  (6.48, 1.13) | 75.57  (165.42, 30.18) | 3.57  (7.87, 1.43) | 0.68  (0.61, 0.75) |
| Independent State of Papua New Guinea | 178.07  (378.13, 76.68) | 18.38  (39.02, 7.95) | 713.70  (1491.61, 304.39) | 27.12  (56.70, 11.58) | 1.01  (0.83, 1.19) |
| Independent State of Samoa | 11.08  (23.10, 4.67) | 30.10  (62.68, 12.65) | 19.73  (42.11, 8.58) | 40.57  (86.43, 17.64) | 0.86  (0.73, 0.99) |
| Ireland | 419.18  (869.06, 181.02) | 47.74  (98.92, 20.63) | 682.05  (1423.32, 297.55) | 59.04  (123.14, 25.76) | 0.64  (0.55, 0.74) |
| Islamic Republic of Afghanistan | 318.37  (678.65, 136.31) | 14.72  (31.30, 6.31) | 1538.47  (3308.63, 660.71) | 21.28  (45.71, 9.15) | 1.73  (1.44, 2.02) |
| Islamic Republic of Iran | 3757.10  (7839.80, 1641.35) | 29.44  (61.28, 12.89) | 9026.95  (18852.13, 3931.99) | 38.99  (81.40, 16.98) | 1.39  (1.03, 1.75) |
| Islamic Republic of Mauritania | 47.21  (101.07, 19.79) | 10.00  (21.47, 4.21) | 151.80  (314.88, 63.61) | 14.07  (29.19, 5.90) | 0.76  (0.57, 0.96) |
| Islamic Republic of Pakistan | 2640.35  (5715.68, 1112.54) | 11.23  (24.25, 4.74) | 8059.31  (17168.07, 3414.60) | 13.26  (28.21, 5.63) | 0.55  (0.38, 0.73) |
| Jamaica | 116.51  (246.62, 48.36) | 19.29  (40.84, 8.01) | 200.93  (426.85, 85.15) | 25.91  (55.07, 10.97) | 0.96  (0.89, 1.04) |
| Japan | 31813.58  (65378.94, 13934.35) | 99.57  (204.41, 43.63) | 26524.60  (54213.49, 11810.08) | 106.56  (217.76, 47.24) | 0.21  (0.16, 0.26) |
| Kingdom of Bahrain | 41.86  (87.50, 17.83) | 35.50  (74.32, 15.14) | 131.66  (278.75, 57.12) | 40.40  (85.50, 17.52) | 0.41  (0.36, 0.45) |
| Kingdom of Belgium | 1264.15  (2653.75, 551.58) | 51.52  (108.19, 22.48) | 1492.18  (3145.12, 661.56) | 60.37  (127.28, 26.77) | 0.39  (0.20, 0.58) |
| Kingdom of Bhutan | 12.96  (27.65, 5.43) | 9.09  (19.31, 3.81) | 33.63  (70.68, 14.20) | 16.13  (33.88, 6.82) | 2.12  (2.00, 2.25) |
| Kingdom of Cambodia | 485.11  (990.01, 206.95) | 19.34  (39.48, 8.26) | 1522.23  (3166.37, 659.21) | 33.48  (69.65, 14.52) | 1.94  (1.89, 1.99) |
| Kingdom of Denmark | 574.99  (1191.97, 253.77) | 43.91  (90.97, 19.34) | 711.88  (1479.67, 315.79) | 56.46  (117.40, 25.01) | 0.79  (0.66, 0.92) |
| Kingdom of Eswatini | 29.79  (62.22, 12.23) | 15.21  (31.74, 6.29) | 59.57  (124.88, 24.76) | 18.68  (39.09, 7.77) | 0.38  (0.13, 0.63) |
| Kingdom of Lesotho | 39.57  (84.72, 16.79) | 10.47  (22.32, 4.45) | 79.21  (169.24, 33.06) | 15.48  (32.93, 6.47) | 1.24  (1.11, 1.37) |
| Kingdom of Morocco | 1680.91  (3537.60, 728.08) | 26.45  (55.73, 11.46) | 3343.84  (6976.78, 1469.91) | 34.57  (72.13, 15.19) | 0.96  (0.92, 1.00) |
| Kingdom of Norway | 509.17  (1059.60, 223.94) | 48.13  (100.21, 21.17) | 665.23  (1375.78, 292.00) | 55.08  (114.09, 24.17) | 0.32  (0.22, 0.43) |
| Kingdom of Saudi Arabia | 1025.72  (2140.93, 444.92) | 31.10  (64.96, 13.52) | 4456.85  (9399.81, 1975.54) | 43.86  (92.77, 19.40) | 1.21  (1.19, 1.23) |
| Kingdom of Spain | 4280.29  (9015.77, 1894.95) | 44.48  (93.69, 19.69) | 5463.69  (11560.49, 2405.93) | 55.87  (117.91, 24.56) | 0.63  (0.50, 0.76) |
| Kingdom of Sweden | 760.24  (1588.58, 334.09) | 37.11  (77.57, 16.30) | 978.01  (2028.13, 429.46) | 44.65  (92.67, 19.60) | 0.48  (0.22, 0.73) |
| Kingdom of Thailand | 5041.26  (10393.17, 2174.78) | 31.30  (64.64, 13.52) | 9579.94  (20155.14, 4206.13) | 59.34  (125.05, 26.04) | 2.20  (2.05, 2.35) |
| Kingdom of the Netherlands | 1716.07  (3553.44, 755.75) | 43.00  (89.01, 18.94) | 1976.69  (4172.82, 856.87) | 53.97  (113.86, 23.38) | 0.68  (0.63, 0.73) |
| Kingdom of Tonga | 7.60  (15.84, 3.27) | 34.12  (71.09, 14.65) | 11.73  (24.39, 5.02) | 46.50  (96.81, 19.90) | 0.69  (0.48, 0.90) |
| Kyrgyz Republic | 59.67  (130.01, 24.24) | 5.65  (12.31, 2.30) | 117.79  (249.88, 48.25) | 6.80  (14.42, 2.78) | 0.46  (0.38, 0.53) |
| Lao People's Democratic Republic | 214.78  (442.57, 92.13) | 22.23  (45.81, 9.54) | 821.82  (1690.29, 356.62) | 41.15  (84.61, 17.86) | 2.22  (2.16, 2.28) |
| Lebanese Republic | 223.40  (458.70, 95.66) | 29.65  (60.99, 12.69) | 576.27  (1199.97, 244.70) | 38.89  (81.19, 16.53) | 0.90  (0.88, 0.91) |
| Malaysia | 1793.49  (3654.73, 772.17) | 39.83  (81.31, 17.16) | 5784.97  (11900.85, 2547.56) | 68.11  (140.09, 30.00) | 1.88  (1.72, 2.03) |
| Mongolia | 27.75  (60.35, 11.31) | 5.40  (11.76, 2.22) | 65.12  (140.44, 27.04) | 7.71  (16.66, 3.20) | 1.26  (1.21, 1.31) |
| Montenegro | 4.37  (9.65, 1.71) | 2.79  (6.17, 1.09) | 5.16  (11.19, 2.03) | 3.60  (7.85, 1.42) | 1.04  (0.95, 1.12) |
| New Zealand | 842.46  (1779.58, 372.97) | 92.98  (196.41, 41.16) | 1133.32  (2378.90, 509.24) | 94.60  (198.63, 42.45) | -0.09  (-0.20, 0.02) |
| North Macedonia | 12.01  (27.03, 4.45) | 2.36  (5.30, 0.87) | 17.27  (37.69, 6.82) | 3.25  (7.10, 1.28) | 1.16  (1.08, 1.23) |
| Northern Mariana Islands | 5.02  (10.72, 2.18) | 35.62  (75.85, 15.44) | 5.15  (10.84, 2.23) | 46.00  (97.06, 19.92) | 0.66  (0.50, 0.82) |
| Palestine | 115.66  (245.24, 48.76) | 25.90  (54.77, 10.93) | 430.47  (912.92, 185.14) | 32.75  (69.41, 14.10) | 0.75  (0.70, 0.80) |
| People's Democratic Republic of Algeria | 1506.92  (3077.02, 628.21) | 25.65  (52.46, 10.73) | 4165.34  (8736.76, 1795.52) | 37.15  (77.88, 16.04) | 1.37  (1.31, 1.43) |
| People's Republic of Bangladesh | 1563.82  (3355.04, 657.24) | 6.36  (13.54, 2.68) | 4813.90  (10018.64, 1997.22) | 10.43  (21.72, 4.33) | 1.84  (1.71, 1.96) |
| People's Republic of China | 44078.48  (92782.77, 18953.65) | 13.70  (28.87, 5.91) | 81337.60  (170556.24, 35351.27) | 25.57  (53.72, 11.12) | 2.09  (1.92, 2.26) |
| Plurinational State of Bolivia | 562.67  (1200.33, 238.70) | 36.81  (78.37, 15.64) | 1542.68  (3353.39, 667.11) | 49.24  (106.97, 21.31) | 0.98  (0.91, 1.06) |
| Portuguese Republic | 1041.30  (2159.32, 458.43) | 41.27  (85.59, 18.17) | 1287.03  (2660.89, 572.26) | 55.90  (115.75, 24.75) | 0.73  (0.55, 0.91) |
| Principality of Andorra | 7.52  (15.56, 3.28) | 49.65  (102.77, 21.67) | 12.07  (25.18, 5.33) | 61.14  (127.49, 26.92) | 0.63  (0.53, 0.74) |
| Principality of Monaco | 3.71  (7.77, 1.61) | 53.09  (111.36, 22.94) | 4.40  (9.30, 1.91) | 62.20  (131.56, 26.99) | 0.46  (0.40, 0.53) |
| Puerto Rico | 286.73  (614.07, 120.60) | 29.93  (64.11, 12.59) | 282.42  (592.86, 121.21) | 38.06  (79.85, 16.35) | 0.82  (0.74, 0.90) |
| Republic of Albania | 20.43  (45.75, 7.68) | 2.43  (5.44, 0.92) | 19.86  (43.95, 7.85) | 3.23  (7.16, 1.28) | 0.87  (0.77, 0.98) |
| Republic of Angola | 154.32  (322.32, 64.70) | 6.74  (14.09, 2.83) | 933.69  (1985.32, 389.32) | 12.16  (25.74, 5.08) | 1.82  (1.67, 1.96) |
| Republic of Armenia | 46.89  (100.04, 18.86) | 5.34  (11.42, 2.15) | 58.57  (126.75, 24.78) | 7.91  (17.08, 3.34) | 1.41  (1.36, 1.47) |
| Republic of Austria | 1262.03  (2609.06, 559.73) | 63.24  (130.77, 28.03) | 1317.39  (2727.72, 566.60) | 66.69  (138.00, 28.68) | -0.09  (-0.18, -0.00) |
| Republic of Azerbaijan | 105.50  (226.80, 42.10) | 5.55  (11.92, 2.22) | 234.38  (510.58, 95.52) | 8.53  (18.66, 3.46) | 1.74  (1.61, 1.86) |
| Republic of Belarus | 86.80  (185.42, 33.84) | 3.40  (7.25, 1.32) | 95.55  (208.29, 38.22) | 4.50  (9.85, 1.79) | 1.11  (1.03, 1.19) |
| Republic of Benin | 82.61  (177.52, 34.76) | 7.50  (16.07, 3.17) | 449.38  (956.69, 189.80) | 13.77  (29.37, 5.83) | 1.83  (1.56, 2.10) |
| Republic of Botswana | 36.06  (76.84, 15.05) | 11.25  (23.97, 4.70) | 120.63  (253.94, 51.79) | 17.63  (37.11, 7.58) | 1.66  (1.45, 1.87) |
| Republic of Bulgaria | 58.57  (132.04, 22.53) | 2.84  (6.43, 1.09) | 51.02  (111.18, 20.30) | 3.62  (7.92, 1.44) | 0.83  (0.79, 0.87) |
| Republic of Burundi | 91.58  (195.41, 38.82) | 7.26  (15.46, 3.09) | 237.16  (504.17, 97.68) | 7.64  (16.24, 3.16) | 0.10  (0.00, 0.19) |
| Republic of Cabo Verde | 6.58  (13.92, 2.71) | 8.29  (17.46, 3.42) | 20.89  (44.85, 8.84) | 13.81  (29.64, 5.84) | 1.44  (1.23, 1.66) |
| Republic of Cameroon | 274.34  (586.74, 113.90) | 11.45  (24.45, 4.78) | 1223.31  (2594.25, 517.64) | 15.48  (32.82, 6.57) | 0.74  (0.64, 0.85) |
| Republic of Chad | 73.06  (156.42, 29.42) | 5.55  (11.82, 2.25) | 326.17  (691.29, 134.74) | 8.47  (17.91, 3.51) | 0.87  (0.61, 1.12) |
| Republic of Chile | 765.61  (1606.03, 323.13) | 21.04  (44.14, 8.87) | 1711.22  (3541.51, 725.05) | 36.08  (74.57, 15.29) | 1.70  (1.39, 2.01) |
| Republic of Colombia | 2470.34  (5290.05, 1058.18) | 28.18  (60.20, 12.06) | 4841.51  (10342.18, 2075.85) | 36.77  (78.64, 15.76) | 0.79  (0.74, 0.85) |
| Republic of Costa Rica | 269.76  (571.29, 116.93) | 34.19  (72.52, 14.83) | 587.47  (1267.48, 249.05) | 45.23  (97.68, 19.21) | 0.79  (0.73, 0.86) |
| Republic of Croatia | 31.30  (68.91, 11.88) | 2.60  (5.73, 0.98) | 31.54  (68.85, 12.13) | 3.53  (7.70, 1.35) | 1.08  (0.98, 1.18) |
| Republic of Cuba | 670.84  (1410.90, 281.53) | 21.73  (45.70, 9.14) | 708.52  (1500.37, 300.23) | 28.88  (61.32, 12.24) | 0.95  (0.90, 1.00) |
| Republic of Cyprus | 78.59  (162.27, 34.31) | 39.60  (81.81, 17.29) | 204.61  (428.71, 90.09) | 56.95  (119.36, 25.10) | 1.29  (1.13, 1.46) |
| Republic of C么te d'Ivoire | 210.97  (451.62, 84.89) | 7.68  (16.40, 3.10) | 851.87  (1831.21, 358.01) | 12.71  (27.25, 5.34) | 1.42  (1.13, 1.71) |
| Republic of Djibouti | 8.33  (17.53, 3.44) | 8.51  (17.90, 3.53) | 46.80  (101.28, 19.66) | 14.51  (31.39, 6.10) | 1.83  (1.69, 1.97) |
| Republic of Ecuador | 1178.59  (2478.92, 513.71) | 46.47  (97.52, 20.24) | 2897.15  (6071.27, 1233.02) | 61.21  (128.17, 26.04) | 0.85  (0.61, 1.08) |
| Republic of El Salvador | 370.31  (785.11, 157.98) | 28.14  (59.51, 11.98) | 704.27  (1506.50, 302.00) | 39.45  (84.34, 16.90) | 1.04  (0.93, 1.14) |
| Republic of Equatorial Guinea | 7.53  (16.08, 3.13) | 7.66  (16.41, 3.20) | 66.03  (137.58, 27.71) | 17.99  (37.44, 7.57) | 3.00  (2.54, 3.45) |
| Republic of Estonia | 13.91  (30.11, 5.32) | 3.64  (7.89, 1.39) | 14.24  (31.22, 5.67) | 5.14  (11.27, 2.04) | 1.41  (1.32, 1.50) |
| Republic of Fiji | 53.38  (110.93, 22.85) | 27.12  (56.29, 11.62) | 94.49  (196.95, 40.80) | 41.37  (86.22, 17.87) | 1.24  (1.09, 1.38) |
| Republic of Finland | 574.31  (1202.22, 254.28) | 45.06  (94.22, 19.97) | 648.29  (1340.58, 286.40) | 57.06  (118.08, 25.17) | 0.69  (0.65, 0.74) |
| Republic of Ghana | 282.88  (601.51, 117.12) | 8.01  (17.04, 3.32) | 1103.21  (2293.09, 467.25) | 12.01  (24.95, 5.09) | 0.88  (0.64, 1.14) |
| Republic of Guatemala | 431.81  (940.30, 181.02) | 23.46  (51.00, 9.84) | 1480.91  (3146.38, 623.75) | 33.45  (71.17, 14.08) | 0.97  (0.85, 1.08) |
| Republic of Guinea | 95.03  (195.52, 39.28) | 6.96  (14.32, 2.89) | 353.20  (768.77, 148.56) | 10.60  (23.01, 4.47) | 1.09  (0.94, 1.24) |
| Republic of Guinea-Bissau | 16.36  (34.16, 6.68) | 7.03  (14.66, 2.87) | 56.28  (119.15, 23.64) | 10.65  (22.50, 4.49) | 1.05  (0.79, 1.33) |
| Republic of Guyana | 36.39  (77.86, 15.29) | 17.59  (37.69, 7.40) | 51.85  (110.91, 22.04) | 25.37  (54.24, 10.80) | 1.18  (1.06, 1.30) |
| Republic of Haiti | 206.27  (442.49, 85.98) | 13.28  (28.52, 5.57) | 571.16  (1225.46, 242.40) | 16.06  (34.43, 6.81) | 0.77  (0.71, 0.83) |
| Republic of Honduras | 241.41  (527.10, 102.08) | 22.75  (49.47, 9.61) | 973.13  (2081.35, 416.55) | 34.01  (72.73, 14.58) | 1.24  (1.12, 1.36) |
| Republic of Iceland | 33.42  (69.55, 14.92) | 51.15  (106.48, 22.84) | 50.93  (106.20, 22.62) | 63.80  (133.01, 28.29) | 0.73  (0.67, 0.78) |
| Republic of India | 23265.88  (49035.91, 10128.56) | 11.49  (24.23, 5.00) | 80390.18  (168395.60, 34609.69) | 21.22  (44.45, 9.14) | 2.29  (2.14, 2.43) |
| Republic of Indonesia | 12331.28  (25410.65, 5308.32) | 25.63  (52.81, 11.05) | 36681.55  (76453.03, 16066.41) | 48.69  (101.59, 21.32) | 2.32  (2.24, 2.40) |
| Republic of Iraq | 1223.46  (2557.98, 526.70) | 29.83  (62.41, 12.84) | 3615.98  (7597.68, 1553.72) | 34.14  (71.71, 14.68) | 0.60  (0.48, 0.72) |
| Republic of Italy | 20152.23  (42414.35, 8914.99) | 141.05  (297.06, 62.38) | 16135.41  (34365.95, 7117.87) | 135.48  (288.85, 59.84) | -0.37  (-0.49, -0.25) |
| Republic of Kazakhstan | 259.11  (564.73, 104.58) | 6.24  (13.62, 2.53) | 411.99  (867.34, 172.24) | 8.72  (18.37, 3.64) | 1.19  (1.16, 1.22) |
| Republic of Kenya | 511.66  (1091.74, 217.10) | 9.99  (21.28, 4.24) | 1632.22  (3447.98, 694.19) | 12.33  (26.07, 5.25) | 0.54  (0.43, 0.65) |
| Republic of Kiribati | 4.32  (9.12, 1.87) | 22.86  (48.23, 9.86) | 10.84  (22.83, 4.71) | 33.73  (71.05, 14.64) | 1.12  (0.90, 1.34) |
| Republic of Korea | 4337.71  (8783.12, 1921.33) | 34.18  (69.28, 15.15) | 6184.12  (12767.82, 2758.64) | 52.77  (109.05, 23.51) | 1.10  (0.76, 1.44) |
| Republic of Latvia | 23.40  (50.91, 9.43) | 3.60  (7.86, 1.45) | 18.17  (39.15, 7.29) | 4.67  (10.08, 1.87) | 1.05  (0.98, 1.12) |
| Republic of Liberia | 44.72  (96.17, 18.57) | 8.00  (17.17, 3.33) | 161.33  (344.03, 68.43) | 11.55  (24.59, 4.90) | 1.35  (1.24, 1.46) |
| Republic of Lithuania | 30.19  (67.60, 11.74) | 3.27  (7.34, 1.27) | 25.72  (55.32, 10.19) | 4.49  (9.63, 1.77) | 1.26  (1.18, 1.34) |
| Republic of Madagascar | 216.80  (467.26, 89.99) | 8.07  (17.42, 3.36) | 680.28  (1421.26, 286.51) | 9.39  (19.61, 3.97) | 0.57  (0.52, 0.62) |
| Republic of Malawi | 240.30  (509.91, 99.71) | 10.57  (22.44, 4.40) | 589.75  (1228.60, 254.46) | 11.77  (24.55, 5.09) | 0.42  (0.30, 0.54) |
| Republic of Maldives | 11.56  (24.04, 4.93) | 24.14  (50.12, 10.33) | 65.07  (141.65, 28.01) | 55.95  (121.75, 24.13) | 3.39  (3.11, 3.68) |
| Republic of Mali | 116.44  (255.70, 48.98) | 6.07  (13.28, 2.55) | 523.28  (1117.42, 220.67) | 9.58  (20.59, 4.07) | 1.24  (1.02, 1.47) |
| Republic of Malta | 43.21  (89.14, 18.84) | 45.70  (94.42, 19.97) | 58.08  (121.59, 25.49) | 61.50  (128.68, 26.91) | 0.89  (0.75, 1.03) |
| Republic of Mauritius | 119.13  (250.13, 51.93) | 39.40  (82.65, 17.19) | 199.33  (418.13, 86.73) | 63.06  (132.36, 27.44) | 1.66  (1.54, 1.77) |
| Republic of Moldova | 32.48  (71.11, 12.75) | 2.87  (6.28, 1.13) | 37.38  (82.67, 15.15) | 4.22  (9.37, 1.70) | 1.49  (1.36, 1.62) |
| Republic of Mozambique | 243.39  (514.78, 101.07) | 7.74  (16.39, 3.22) | 885.63  (1864.33, 371.58) | 11.64  (24.38, 4.90) | 1.32  (1.23, 1.42) |
| Republic of Namibia | 37.15  (81.00, 15.75) | 10.99  (23.86, 4.66) | 97.99  (204.56, 42.37) | 14.74  (30.82, 6.37) | 0.88  (0.73, 1.02) |
| Republic of Nauru | 0.71  (1.51, 0.30) | 29.15  (61.59, 12.46) | 1.21  (2.53, 0.53) | 42.19  (88.37, 18.51) | 1.06  (0.99, 1.12) |
| Republic of Nicaragua | 236.41  (502.20, 100.15) | 25.99  (55.11, 11.00) | 673.13  (1450.62, 289.95) | 36.92  (79.53, 15.91) | 1.03  (0.94, 1.13) |
| Republic of Niue | 0.16  (0.33, 0.07) | 31.90  (66.33, 13.46) | 0.18  (0.38, 0.08) | 48.28  (100.07, 20.77) | 1.26  (1.10, 1.42) |
| Republic of Palau | 1.38  (2.96, 0.59) | 33.37  (71.72, 14.38) | 1.76  (3.62, 0.76) | 48.13  (99.42, 20.79) | 1.03  (0.87, 1.20) |
| Republic of Panama | 147.12  (320.49, 62.11) | 23.83  (51.91, 10.04) | 406.68  (889.10, 174.67) | 38.02  (83.14, 16.33) | 1.35  (1.25, 1.44) |
| Republic of Paraguay | 60.70  (129.82, 24.64) | 6.47  (13.83, 2.63) | 191.79  (410.55, 78.39) | 10.07  (21.58, 4.12) | 1.65  (1.59, 1.71) |
| Republic of Peru | 2028.87  (4447.75, 880.65) | 37.31  (81.61, 16.22) | 5106.65  (10892.15, 2205.91) | 52.84  (112.75, 22.84) | 1.22  (1.18, 1.26) |
| Republic of Poland | 406.51  (848.89, 166.00) | 4.31  (9.01, 1.76) | 393.59  (821.85, 171.87) | 4.50  (9.42, 1.96) | -0.01  (-0.08, 0.05) |
| Republic of Rwanda | 154.57  (327.71, 64.58) | 9.50  (20.10, 3.98) | 421.02  (891.83, 176.97) | 11.92  (25.24, 5.01) | 0.78  (0.71, 0.86) |
| Republic of San Marino | 3.18  (6.73, 1.40) | 50.82  (107.45, 22.38) | 4.22  (8.95, 1.83) | 60.13  (127.65, 26.05) | 0.50  (0.42, 0.59) |
| Republic of Senegal | 158.30  (343.39, 66.43) | 9.17  (19.86, 3.85) | 452.79  (961.51, 189.93) | 11.55  (24.57, 4.86) | 0.51  (0.39, 0.64) |
| Republic of Serbia | 56.94  (125.55, 21.60) | 2.44  (5.38, 0.92) | 67.01  (147.20, 26.23) | 3.31  (7.29, 1.29) | 1.11  (1.05, 1.17) |
| Republic of Seychelles | 7.69  (15.86, 3.31) | 42.19  (86.86, 18.21) | 14.29  (29.72, 6.18) | 59.28  (123.37, 25.63) | 1.07  (1.01, 1.13) |
| Republic of Sierra Leone | 69.17  (143.02, 28.78) | 6.86  (14.21, 2.87) | 266.60  (566.07, 111.20) | 11.66  (24.77, 4.87) | 1.51  (1.36, 1.66) |
| Republic of Singapore | 371.28  (748.92, 165.26) | 39.00  (78.72, 17.38) | 932.69  (1938.99, 422.14) | 62.48  (130.21, 28.20) | 1.53  (1.38, 1.68) |
| Republic of Slovenia | 13.13  (28.53, 5.01) | 2.62  (5.70, 1.00) | 15.35  (32.96, 6.20) | 3.66  (7.92, 1.47) | 1.14  (1.03, 1.25) |
| Republic of South Africa | 1494.66  (3194.96, 641.48) | 15.42  (32.90, 6.63) | 3046.93  (6459.75, 1290.56) | 19.57  (41.51, 8.29) | 0.89  (0.82, 0.96) |
| Republic of South Sudan | 113.90  (247.29, 46.23) | 8.78  (18.99, 3.58) | 225.00  (483.55, 93.86) | 9.76  (20.91, 4.08) | 0.20  (0.09, 0.30) |
| Republic of Sudan | 804.83  (1733.70, 345.00) | 17.08  (36.75, 7.33) | 3405.57  (7179.22, 1486.86) | 29.99  (63.17, 13.10) | 1.93  (1.76, 2.09) |
| Republic of Suriname | 19.29  (40.83, 8.14) | 19.66  (41.63, 8.31) | 38.66  (83.11, 16.63) | 26.73  (57.49, 11.50) | 0.99  (0.95, 1.03) |
| Republic of Tajikistan | 55.32  (120.03, 22.07) | 4.56  (9.87, 1.83) | 152.40  (331.15, 60.92) | 5.94  (12.91, 2.38) | 0.93  (0.86, 1.00) |
| Republic of the Congo | 52.54  (109.59, 21.55) | 9.34  (19.50, 3.85) | 186.11  (392.02, 79.24) | 12.99  (27.31, 5.54) | 0.88  (0.72, 1.04) |
| Republic of the Gambia | 17.71  (37.49, 7.38) | 7.77  (16.43, 3.24) | 71.77  (151.21, 29.63) | 11.60  (24.44, 4.82) | 1.03  (0.87, 1.19) |
| Republic of the Marshall Islands | 1.92  (4.16, 0.82) | 19.59  (42.24, 8.41) | 4.88  (10.20, 2.11) | 32.97  (68.85, 14.25) | 1.46  (1.28, 1.64) |
| Republic of the Niger | 111.49  (235.04, 45.25) | 6.42  (13.46, 2.62) | 454.04  (960.85, 186.74) | 8.57  (18.19, 3.55) | 0.83  (0.69, 0.97) |
| Republic of the Philippines | 4307.60  (8911.37, 1818.28) | 27.85  (57.60, 11.78) | 14010.92  (29270.74, 5964.60) | 47.69  (99.66, 20.33) | 2.15  (2.02, 2.29) |
| Republic of the Union of Myanmar | 2146.45  (4403.33, 916.43) | 20.50  (42.16, 8.78) | 6367.45  (13352.47, 2759.38) | 42.16  (88.43, 18.26) | 2.77  (2.63, 2.91) |
| Republic of Trinidad and Tobago | 62.37  (132.35, 26.38) | 20.12  (42.62, 8.52) | 96.66  (202.18, 41.22) | 28.70  (60.07, 12.25) | 1.29  (1.15, 1.44) |
| Republic of Tunisia | 532.87  (1113.08, 226.00) | 25.78  (53.83, 10.96) | 1078.16  (2265.33, 469.26) | 35.39  (74.51, 15.40) | 1.19  (1.14, 1.24) |
| Republic of Turkey | 3510.66  (7365.23, 1524.74) | 24.42  (51.21, 10.64) | 7111.86  (14976.91, 3084.03) | 33.01  (69.52, 14.31) | 1.06  (0.99, 1.14) |
| Republic of Uganda | 345.53  (724.85, 142.97) | 8.97  (18.83, 3.74) | 1154.50  (2418.44, 483.16) | 11.06  (23.21, 4.62) | 0.67  (0.62, 0.72) |
| Republic of Uzbekistan | 289.81  (621.00, 115.71) | 5.87  (12.55, 2.35) | 733.15  (1586.84, 296.32) | 8.20  (17.77, 3.32) | 1.12  (0.99, 1.25) |
| Republic of Vanuatu | 7.94  (16.77, 3.39) | 22.50  (47.49, 9.62) | 26.46  (56.24, 11.45) | 33.61  (71.45, 14.55) | 1.21  (1.15, 1.26) |
| Republic of Yemen | 489.61  (1023.61, 212.34) | 17.76  (36.99, 7.71) | 1843.44  (3931.23, 790.35) | 21.97  (46.82, 9.44) | 0.99  (0.86, 1.12) |
| Republic of Zambia | 217.97  (474.33, 90.83) | 11.84  (25.70, 4.94) | 720.23  (1523.68, 300.82) | 14.54  (30.71, 6.09) | 0.47  (0.38, 0.55) |
| Republic of Zimbabwe | 302.33  (639.71, 129.32) | 12.57  (26.53, 5.39) | 552.24  (1184.47, 232.22) | 13.55  (28.93, 5.71) | -0.15  (-0.34, 0.05) |
| Romania | 136.57  (296.37, 51.47) | 2.43  (5.28, 0.92) | 138.80  (304.75, 54.36) | 3.46  (7.60, 1.34) | 1.24  (1.19, 1.30) |
| Russian Federation | 1344.72  (2857.41, 553.89) | 3.58  (7.62, 1.47) | 1588.35  (3339.34, 657.28) | 4.63  (9.76, 1.91) | 1.00  (0.95, 1.04) |
| Saint Kitts and Nevis | 2.30  (4.94, 0.98) | 22.68  (48.53, 9.61) | 4.77  (10.00, 2.06) | 30.75  (64.52, 13.24) | 0.90  (0.82, 0.98) |
| Saint Lucia | 6.51  (13.64, 2.72) | 18.83  (39.54, 7.89) | 11.45  (24.40, 4.90) | 25.26  (54.00, 10.81) | 0.67  (0.53, 0.82) |
| Saint Vincent and the Grenadines | 4.61  (9.84, 1.92) | 17.25  (36.60, 7.25) | 7.17  (15.44, 3.08) | 25.92  (55.88, 11.11) | 1.36  (1.26, 1.46) |
| Slovak Republic | 33.16  (72.51, 12.52) | 2.50  (5.46, 0.94) | 43.94  (98.10, 17.38) | 3.49  (7.81, 1.38) | 1.08  (1.04, 1.12) |
| Socialist Republic of Viet Nam | 3289.05  (6804.88, 1417.97) | 19.17  (39.67, 8.26) | 9840.91  (20663.32, 4246.68) | 38.41  (80.56, 16.57) | 2.76  (2.58, 2.94) |
| Solomon Islands | 14.48  (30.55, 6.34) | 19.47  (41.14, 8.53) | 52.45  (112.44, 22.66) | 30.58  (65.47, 13.20) | 1.23  (0.98, 1.48) |
| State of Eritrea | 50.78  (105.52, 21.30) | 6.47  (13.44, 2.73) | 160.41  (338.27, 65.33) | 9.67  (20.46, 3.94) | 1.26  (1.07, 1.46) |
| State of Israel | 508.00  (1066.63, 224.77) | 41.71  (87.48, 18.49) | 1189.15  (2463.33, 515.51) | 53.71  (111.28, 23.28) | 0.72  (0.63, 0.80) |
| State of Kuwait | 160.62  (339.46, 69.27) | 37.98  (80.08, 16.40) | 663.43  (1395.63, 288.38) | 45.70  (96.94, 19.84) | 0.79  (0.73, 0.85) |
| State of Libya | 312.83  (655.93, 133.64) | 34.01  (71.36, 14.55) | 742.49  (1550.57, 314.60) | 37.52  (78.49, 15.88) | 0.47  (0.41, 0.52) |
| State of Qatar | 30.31  (63.50, 12.77) | 37.91  (79.43, 16.02) | 248.80  (514.52, 106.33) | 44.75  (92.82, 19.17) | 0.53  (0.49, 0.57) |
| Sultanate of Oman | 83.98  (177.40, 36.08) | 24.56  (51.77, 10.56) | 413.45  (871.72, 176.85) | 40.53  (85.34, 17.35) | 1.74  (1.65, 1.83) |
| Swiss Confederation | 843.32  (1757.44, 367.95) | 47.54  (99.17, 20.70) | 1045.80  (2160.38, 446.93) | 53.42  (110.24, 22.77) | 0.41  (0.39, 0.42) |
| Syrian Arab Republic | 713.86  (1497.89, 308.56) | 25.63  (53.64, 11.09) | 1301.67  (2756.82, 562.31) | 33.89  (71.57, 14.66) | 0.98  (0.90, 1.07) |
| Taiwan (Province of China) | 1417.11  (3042.07, 594.60) | 25.49  (54.74, 10.71) | 2300.98  (4701.02, 998.15) | 41.06  (83.72, 17.78) | 1.78  (1.70, 1.86) |
| Togolese Republic | 62.10  (133.26, 26.03) | 7.27  (15.59, 3.06) | 241.04  (515.86, 100.73) | 11.14  (23.85, 4.67) | 1.10  (0.93, 1.27) |
| Tokelau | 0.09  (0.20, 0.04) | 26.70  (56.24, 11.44) | 0.14  (0.29, 0.06) | 43.62  (90.89, 19.36) | 1.48  (1.34, 1.63) |
| Turkmenistan | 51.22  (110.92, 20.49) | 5.74  (12.40, 2.31) | 104.79  (226.51, 43.29) | 8.33  (18.00, 3.44) | 1.25  (1.23, 1.28) |
| Tuvalu | 0.61  (1.25, 0.26) | 24.90  (50.93, 10.71) | 1.15  (2.43, 0.51) | 39.38  (83.33, 17.55) | 1.28  (1.11, 1.46) |
| Ukraine | 435.37  (917.30, 173.31) | 3.41  (7.20, 1.35) | 419.55  (891.09, 172.58) | 4.08  (8.74, 1.66) | 0.77  (0.70, 0.85) |
| Union of the Comoros | 11.64  (24.72, 4.80) | 11.03  (23.44, 4.57) | 25.78  (53.57, 10.85) | 13.17  (27.38, 5.55) | 0.34  (0.18, 0.50) |
| United Arab Emirates | 108.68  (226.73, 47.08) | 31.10  (64.90, 13.49) | 688.40  (1439.84, 298.76) | 40.86  (85.53, 17.76) | 0.84  (0.72, 0.96) |
| United Kingdom of Great Britain and Northern Ireland | 7499.80  (15569.87, 3316.25) | 52.51  (109.09, 23.20) | 10022.28  (20926.13, 4440.61) | 65.39  (136.60, 28.94) | 0.47  (0.34, 0.60) |
| United Mexican States | 12921.74  (27494.39, 5669.86) | 58.76  (125.04, 25.71) | 20531.82  (43801.31, 8936.54) | 58.71  (125.24, 25.55) | -0.55  (-0.79, -0.30) |
| United Republic of Tanzania | 616.73  (1282.59, 257.06) | 10.26  (21.26, 4.29) | 1861.29  (3919.40, 812.28) | 12.52  (26.39, 5.47) | 0.62  (0.53, 0.70) |
| United States of America | 36560.40  (75129.26, 16025.42) | 53.91  (110.67, 23.64) | 51044.37  (101979.11, 23334.71) | 67.35  (134.59, 30.78) | -0.60  (-1.13, -0.06) |
| United States Virgin Islands | 8.27  (17.70, 3.48) | 29.05  (62.13, 12.22) | 6.17  (13.06, 2.65) | 36.50  (77.43, 15.64) | 0.70  (0.60, 0.81) |

Abbreviations: ASDR, age standardized DALYs rate; EAPC, estimated annual percentage change; SDI, socio-demographic index; UI, uncertainty interval; CI, confidence interval.
