# Supplementary material for: Global burden of polycystic ovary syndrome in women of reproductive age, 1990–2021: Analysis of the global burden of disease study 2021 with projections to 2050
Source: PLoS One. 2025 Oct 7;20(10):e0333000. doi: 10.1371/journal.pone.0333000 (PMC12503323; doi:10.1371/journal.pone.0333000)
Supplement: Attachment — Submitted filename: Response Letter.docx [file pone.0333000.s020.docx]

**Response Letter**

**Title:** Global burden of polycystic ovary syndrome in women of reproductive age, 1990-2021: Analysis of the Global Burden of Disease Study 2021 with projections to 2050

**Authors:** Xinkuo Zheng*, Meishen Liu, Zhaohui Bai, Ling Wu, Lili Geng, Yun Shen, Jing Na, Haonan Qiu, Yalin Xi*

**Manuscript Number:** PONE-D-25-17138

Dear Dr. Editor,

Please find enclosed the revised version of our previous submission entitled "Global burden of polycystic ovary syndrome in women of reproductive age, 1990-2021: Analysis of the Global Burden of Disease Study 2021 with projections to 2050" with manuscript number: PONE-D-25-17138.

We wish to extend our deepest gratitude to the esteemed Editor and the dedicated editorial team for your invaluable time and meticulous efforts in handling our manuscript amidst your demanding schedules. Your exceptional professionalism and insightful guidance have been instrumental in enhancing the manuscript, especially amidst your demanding schedules.

We are equally indebted to the distinguished reviewers for their thoughtful critique and generous recognition of our research. Their profound expertise and constructive suggestions have not only strengthened our manuscript but also provided us with truly enlightening perspectives. It is an honor to receive such discerning feedback from scholars of their expertise.

In this revised manuscript, we have meticulously addressed all comments. Detailed point-by-point responses to the journal requirements and reviewers' feedback are provided below.

Sincerely,

Xinkuo Zheng

Email: [xinkuo1994@hotmail.com](mailto:xinkuo1994@hotmail.com)

***Response to Journal Requirements***

**Comment 1:** Please ensure that your manuscript meets PLOS ONE's style requirements, including those for file naming.

**Response:** We have downloaded the PLOS ONE style templates and carefully revised our manuscript to match the formatting requirements. For your convenience, all changes have been clearly marked with comments in the manuscript. We have also renamed the uploaded figures and tables according to the template specifications. Kindly review these updates at your convenience.

**Comment 2:** Your ethics statement should only appear in the Methods section of your manuscript. If your ethics statement is written in any section besides the Methods, please delete it from any other section.

**Response:** Thank you very much for your guidance. We duly noted that the ethics statement appeared not only in the Materials and Methods section but also at the end of the manuscript. Therefore, per your suggestion, we have removed the ethics statement from the end of the manuscript (line 418). Kindly review this adjustment.

**Comment 3:** We note that of Figure 2, S2 & S4 Figure in your submission contain [map/satellite] images which may be copyrighted. All PLOS content is published under the Creative Commons Attribution License (CC BY 4.0), which means that the manuscript, images, and Supporting Information files will be freely available online, and any third party is permitted to access, download, copy, distribute, and use these materials in any way, even commercially, with proper attribution. For these reasons, we cannot publish previously copyrighted maps or satellite images created using proprietary data, such as Google software (Google Maps, Street View, and Earth).

**Response:** Thank you for raising questions regarding the map images in Figure 2, S2 & S4 Figure. Please find our responses below:

a) Source of Map Images:

The map images in article were created using the rnaturalearth R package, which provides access to Natural Earth map data. This data is open-source and available at https://github.com/ropensci/rnaturalearth.

b) Copyright Status:

To our knowledge, the map data from Natural Earth provided through the rnaturalearth package is in the public domain and therefore not subject to copyright restrictions. Natural Earth map data is widely used for world mapping and is explicitly designed for open, unrestricted use.

c) Permission for Use:

Since the map images derive from open-source public domain data provided by Natural Earth, we believe no additional copyright permission is required. However, we can include a statement attributing Natural Earth as the data source if needed.

**Comment 4:** If the reviewer comments include a recommendation to cite specific previously published works, please review and evaluate these publications to determine whether they are relevant and should be cited. There is no requirement to cite these works unless the editor has indicated otherwise.

**Response:** Thank you so much for your kind reminder. We would like to note that the reviewers have not raised any requests for citing specific references so far.

**Comment 5:** Please review your reference list to ensure that it is complete and correct. If you have cited papers that have been retracted, please include the rationale for doing so in the manuscript text, or remove these references and replace them with relevant current references. Any changes to the reference list should be mentioned in the rebuttal letter that accompanies your revised manuscript. If you need to cite a retracted article, indicate the article’s retracted status in the References list and also include a citation and full reference for the retraction notice.

**Response:** Thank you very much for your review comments and suggestions. We have verified all references and confirmed that none contain retracted papers. Our reference formatting strictly matches the first citation style in the journal template. We modified the formatting of Reference [1] and [30] to comply with the template requirements (lines 426 and 508). Besides, we have cited several references in the newly added content, with the following reference numbers: [26], [27], [28]. No other references were altered.

***Response to Reviewer 1***

Dear Dr. Reviewer 1,

Please find enclosed the revised version of our previous submission entitled "Global burden of polycystic ovary syndrome in women of reproductive age, 1990-2021: Analysis of the Global Burden of Disease Study 2021 with projections to 2050" with manuscript number: PONE-D-25-17138.

We are very grateful that you took valuable time out of your busy schedule to review our manuscript. We are also honored to have received recognition from such a distinguished international scholar. Your encouragement is a strong motivation for us to further improve this study. Furthermore, your deep knowledge, sharp insights, and rigorous approach will have a significant influence on our future research careers.

In this revised manuscript, we have meticulously addressed all the comments. Detailed point-by-point responses to the feedback from you are provided below.

Finally, we would like to once again express our sincere gratitude and wish you all the best in your research, professional endeavors, and personal life.

Sincerely,

Xinkuo Zheng

**Comment 1:** Software Concerns: The manuscript states that "All statistical analyses and visualizations were performed using R software (version 4.4.2) and JD_GBDR (V2.36, Jingding Medical Technology Co., Ltd)". Please clarify the specific role of the JD_GBDR software in the analysis. Consider to provide sufficient detail for transparency or confirm if the analyses can be fully replicated using only R and its standard packages.

**Response:** Thank you so much for your interest in our statistical software. JD_GBDR is an R-based graphical analysis tool designed for Global Burden of Disease (GBD) data. Functioning as an integrated platform within the R environment, it provides a unified interface with modular architecture to facilitate GBD data loading, processing, visualization, and predictive modeling. All analytical procedures are performed using publicly available R package functions. Core computations rely exclusively on the following open-source R packages (beyond base R, including but not limited to):

| library(DT) | library(readxl) | library(tidyverse) | library(tidyr) |
| --- | --- | --- | --- |
| library(rnaturalearth) | library(rnaturalearthdata) | library(sf) | library(scales) |
| library(broom) | library(ggrepel) | library(patchwork) | library(segmented) |
| library(readr) | library(ggpubr) | library(forecast) | library(tseries) |
| library(INLA) | library(BAPC) | library(vroom) | library(car) |
| library(MASS) | library(mgcv) | library(splines) | library(httr) |
| library(jsonlite) |  |  |  |

This implementation ensures complete reproducibility of results through standard R software.

**Comment 2:** Figure Clarity: In the reviewed PDF version, the legibility of some figures, notably Figure 5, is suboptimal. Please ensure that all figures are provided at a sufficiently high resolution in the final submission.

**Response:** Thank you very much for identifying the figure clarity issues. Your suggestions will significantly improve the readability of our manuscript. Following your advice, we have checked all figures and adjusted them to 600 dpi resolution. Additionally, in accordance with the guidelines for resubmitting the figure files, we have uploaded the figures to be inserted in the main text (Figs 1-5) to the PACE digital diagnostic tool and downloaded the adjusted figures processed by the tool. Please view them in the separately uploaded figure files.

***Response to Reviewer 2***

Dear Dr. Reviewer 2,

Please find enclosed the revised version of our previous submission entitled "Global burden of polycystic ovary syndrome in women of reproductive age, 1990-2021: Analysis of the Global Burden of Disease Study 2021 with projections to 2050" with manuscript number: PONE-D-25-17138.

We are very grateful that you took valuable time out of your busy schedule to review our manuscript. We are also honored to have received recognition from such a distinguished international scholar. Your encouragement is a strong motivation for us to further improve this study. Furthermore, your deep knowledge, sharp insights, and rigorous approach will have a significant influence on our future research careers.

In this revised manuscript, we have meticulously addressed all the comments. Detailed point-by-point responses to the feedback from you are provided below.

Finally, we would like to once again express our sincere gratitude and wish you all the best in your research, professional endeavors, and personal life.

Sincerely,

Xinkuo Zheng

**Comment 1:** Suggest to spell out EAPC in the abstract.

**Response:** We sincerely appreciate your highly thorough review of our manuscript. As per your suggestion, we have spelled out EAPC in the Abstract. Please refer to Line 34 of the manuscript for verification.

**Comment 2:** Line 281: repetition of “but also”.

**Response:** We sincerely appreciate your valuable review comments. In accordance with your comment, we have deleted a redundant instance of 'but also'. Please see Line 270 of the manuscript for reference.

**Comment 3:** Suggest to discuss the impact of exclusive used of the NIH criteria in GBD that might affect the actual incidence & prevalence of PCOS in countries where Rotterdam or AE-PCOS criteria are used.

**Response:** Thank you for your insightful review and emphasis on the diagnostic criteria variations in PCOS. As suggested, we have added a paragraph in the Discussion section addressing how the NIH criteria in GBD may affect the actual incidence & prevalence of PCOS in countries using Rotterdam or AE-PCOS criteria. The new content can be found at Line 290 of the revised manuscript. For your convenience, we also enclose it below.

"The exclusive adoption of NIH 1990 criteria in the GBD study may substantially underestimate the true burden of PCOS in regions where Rotterdam or AE-PCOS criteria are routinely applied. Extensive data indicate that diagnostic criteria critically determine PCOS prevalence estimates: NIH-based rates range 4%-6.6% globally, whereas Rotterdam criteria yield prevalences of 6%-21% [3]. This discrepancy primarily arises from Rotterdam’s inclusion of broader phenotypes (e.g., normoandrogenic phenotypes with ovulatory dysfunction and polycystic ovarian morphology) [3,26]. Crucially, country-specific studies in Turkey and Denmark demonstrate Rotterdam-based prevalence rates reaching 2-3 times NIH-based figures (e.g., 19.9% vs 6.1% in Turkey) [27]. GBD’s sole reliance on NIH criteria thus introduces dual biases: First, it systematically underestimates disease magnitude in regions predominantly using Rotterdam criteria. Second, it excludes large subgroups of mild or non-hyperandrogenic phenotypes, distorting public health resource allocation [28]. We recommend incorporating sensitivity analyses across diagnostic criteria through stratified burden reporting to better reflect global epidemiological variations."

Please note that we have added three new references in this paragraph, as follows:

26.	Broekmans FJ, Knauff EA, Valkenburg O, Laven JS, Eijkemans MJ, Fauser BC. PCOS according to the Rotterdam consensus criteria: Change in prevalence among WHO-II anovulation and association with metabolic factors. BJOG. 2006 Oct;113(10):1210-7.

27.	Yildiz BO, Bozdag G, Yapici Z, Esinler I, Yarali H. Prevalence, phenotype and cardiometabolic risk of polycystic ovary syndrome under different diagnostic criteria. Hum Reprod. 2012 Oct;27(10):3067-73.

28.	Tehrani FR, Rashidi H, Khomami MB, Tohidi M, Azizi F. The prevalence of metabolic disorders in various phenotypes of polycystic ovary syndrome: a community based study in Southwest of Iran. Reprod Biol Endocrinol. 2014 Sep 16;12:89.

**Comment 4:** The manuscript mentioned the impact of non-modifiable risk factors such as sedentary lifestyles and insulin resistance. suggest to support this using quantitative linkage or risk attribution analysis.

**Response:** We sincerely appreciate the reviewer's valuable suggestion to strengthen our analysis through quantitative linkage or risk attribution methods for non-modifiable factors like sedentary lifestyles and insulin resistance. While the manuscript cites existing studies supporting the impact of these factors on PCOS, our thorough consultation of the GBD database confirms that sedentary lifestyles and insulin resistance are not systematically quantified in its current PCOS metadata framework. Due to these fundamental data constraints, we regret being unable to incorporate the requested analysis at this stage. Should such data become accessible in future studies, we will prioritize this important validation.

**Comment 5:** With the projected increased burden of PCOS by the year 2050, suggest to add future studies that could evaluate intervention effectiveness.

**Response:** We appreciate the reviewer's suggestion regarding future intervention studies in light of the projected rise in PCOS burden by 2050. We have expanded the Discussion section to include perspectives on future PCOS research directions. The newly added content can be found at Line 375 of the revised manuscript. For your convenience, we reproduce the additional text below:

"Looking ahead to 2050 when PCOS burden is projected to increase substantially, critical knowledge gaps remain regarding optimal intervention strategies, particularly concerning phenotype-specific approaches and scalable solutions for diverse populations; future studies should therefore prioritize three key areas: (1) large-scale randomized trials comparing lifestyle modifications, pharmacotherapy, and their combinations stratified by Rotterdam phenotypes, (2) cost-effectiveness analyses of early intervention in adolescent populations showing early PCOS markers, and (3) implementation research testing digital health platforms for long-term management in low-resource settings, as these approaches would not only address current evidence limitations but also provide actionable data for healthcare systems preparing for the anticipated rise in PCOS prevalence."

**Comment 6:** Suggest potential policy level recommendations based on the different SDI regions.

**Response:** We sincerely appreciate the reviewer's insightful comments regarding policy-level recommendations across SDI regions. In response, we have incorporated targeted policy proposals tailored to different SDI levels, which can be found at Line 330 of the revised manuscript. For the reviewer's convenience, the newly added text is reproduced below:

"These findings underscore the need for tailored policy interventions across SDI regions to address the varying PCOS burden effectively. In high-SDI regions, policies should prioritize comprehensive PCOS screening programs integrated into routine reproductive healthcare, coupled with public health campaigns targeting modifiable risk factors such as physical inactivity and metabolic disorders through workplace wellness initiatives and nutritional education. For middle-SDI regions, where the most rapid increase in burden was observed, health systems should focus on capacity-building for early diagnosis through primary care physician training and standardized diagnostic protocols, while investing in cost-effective interventions such as community-based lifestyle modification programs. Low-SDI regions would benefit most from foundational healthcare strengthening, including the deployment of simplified diagnostic tools (e.g., mobile ultrasound units) and task-shifting strategies to enable frontline healthcare workers to identify PCOS cases. Across all SDI levels, longitudinal research funding should be allocated to clarify the interplay between socioeconomic development and PCOS epidemiology, with particular attention to distinguishing healthcare access limitations from true biological risk factors in observed disparities, as this will be critical for refining global guidelines and ensuring equitable resource allocation aligned with the UN’s Sustainable Development Goals for women’s health."
